# Prevalence of Mobile Phones and Factors Influencing Usage by Caregivers of Young Children in Daily Life and for Health Care in Rural China: A Mixed Methods Study

**DOI:** 10.1371/journal.pone.0116216

**Published:** 2015-03-19

**Authors:** Michelle Helena van Velthoven, Ye Li, Wei Wang, Li Chen, Xiaozhen Du, Qiong Wu, Yanfeng Zhang, Igor Rudan, Josip Car

**Affiliations:** 1 Global eHealth Unit, Department of Primary Care and Public Health, Imperial College London, London, United Kingdom; 2 Department of Integrated Early Childhood Development, Capital Institute of Paediatrics, Beijing, China; 3 Centre for Population Health Sciences and Global Health Academy, University of Edinburgh Medical School, Edinburgh, United Kingdom; Brown University, UNITED STATES

## Abstract

**Introduction:**

To capitalise on mHealth, we need to understand the use of mobile phones both in daily life and for health care.

**Objective:**

To assess the prevalence and factors that influence usage of mobile phones by caregivers of young children.

**Materials and Methods:**

A mixed methods approach was used, whereby a survey (N=1854) and semi-structured interviews (N=17) were conducted concurrently. The quantitative and qualitative data obtained were compared and integrated. Participants were caregivers of young children in Zhao County, Hebei Province, China.

**Results:**

Four main themes were found: (i) trends in mobile phone ownership; (ii) usage of mobile phone functions; (iii) factors influencing replying to text messages; and (iv) uses of mobile phones for health care. The majority of 1,854 survey participants (1,620; 87.4%) used mobile phones, but usage was much higher among mothers (1,433; 92.6%) and fathers (41; 100.0%) compared to grandparents (142; 54.6%). Parents were able to send text messages, grandparents often not. Factors influencing the decision to reply to text messages in daily life were checking the mobile phone, trusting the sender, emotion or feeling when receiving a text message, the importance of replying and ease of use of text messages. Of 1,620 survey participants who used a mobile phone, about one in four (432; 26.7%) had used it for health care in the past three months and most (1,110; 93.5%) of 1,187 who had not wished to use their phone to receive health information.

**Conclusion:**

We found that usage of mobile phones is high, several factors influencing usage and an interest of caregivers to use phones for health care in Zhao County, rural China, which can be used to inform studies in settings with similar characteristics. Future work needs to assess factors influencing mobile phone usage in-depth to optimize experiences of users for specific mHealth-based interventions.

## Introduction

By the end of 2014, an estimated 78% of the 6.9 billion mobile phone subscriptions will be in low- and middle-income countries [1]. China ranked first in the world’s mobile markets with an estimated 1.2 billion mobile phone subscriptions, which accounts for approximately 92% of the population in 2014[2]. Mobile phone text messaging is very popular function of mobile phones. In 2013, an estimated 431 billion text messages were sent inChina, where sending a text message usually costs ¥0.10 (approximately£0.01, € 0.12, US$ 0.16) and receiving a text message is free [3].

As mobile phones have become part of daily life and many people carry them at all times, they provide an opportunity to reach those who were previously hard to reach via traditional modes of communication. This is particularly relevant to people without access to high quality health care in low- and middle-income countries [4–7]. Mobile phones can improve communication and delivery of information over long distances between health care providers, patients and healthy individuals [8]. This can potentiallyimprove the efficiency and effectiveness of inadequate health care systems and ultimately result in benefits for people [9,10].

Mobile health, or mHealth, stands for the usage of mobile information and communication technologies for health-related purposes. An understanding of usage of mobile phones is a precondition for effective mHealth-based interventions. Although mobile phones are widely used, there may be differences in access and usagein different settings. Knowledge of factors influencing mobile phone usage is needed to optimize experiences of users of mHealth-based interventions [5,11–14]. In addition, exploring the natural role of mobile phones in health care can facilitate integration of mHealth-based interventions in health systems and optimize health benefits [5].

However, assessing usage of mobile phones by populations has been under-researched [15,16]. For example in Kenya, where most mHealth-based trials in low- and-middle income countries have taken place [17–21], only two recent quantitative studies assessed usage of mobile phones [22,23]. In China, only one mixed methods study explored usage of mobile phones among pulmonary tuberculosis patients in Chongqing Province in Western China [24].

In the specificrural context of our study, Zhao County, Hebei Province in Northern China, previous observations showed that mobile phones were commonly used [25]. However, information on how exactly caregivers used their mobile phones in their daily lives and for health care was unknown. Therefore, we assessed the prevalence and factors influencing the usage of mobile phones by caregivers in Zhao County. The results of this study were used to inform mHealth-based studies in this setting on collecting health-related information via text messages [26–30].

## Materials and Methods

A mixed methods study was undertaken, whereby perspectives of both quantitative and qualitative approaches are used and data are purposely combined. These approaches complement each other by making use of the strengths of both and answer questions that are inadequately answered by a single approach [31,32]. A survey and semi-structured interviews were conducted concurrently. The survey assessed prevalence of usage of mobile phones, both in daily life and for health care. Semi-structured interviews explored factors influencing usage of mobile phones, and how mobile phones were used for health care. The quantitative and qualitative data obtained from these strategies were given equal weight and were compared and integrated. The Ethical Committee of the Capital Institute of Pediatrics in Beijing provided ethical approval. All participants provided written informed consent prior to their inclusion in the study. Part of the methodology of this study was previously reported elsewhere [26].

### Study setting and sample

Both the survey and the semi-structured interviews took place in Zhao County, Hebei Province, China in January 2013. Zhao County is located 280 kilometers south of Beijing. Zhao County has a total population of 571,000, with 518,000 people (90.7%) living in rural areas. The socioeconomic development of Zhao County is similar to Hebei Province, similar to the national average. The female illiteracy rate is low (3.8%) and the main ethnic group is Han (99.9%) (data from 2010 provided by the Zhao County Statistics Bureau, unpublished).

**Survey.** The survey was part of a randomised controlled trial aiming to assess the effectiveness of infant feeding information disseminated via QQ (a popular Chinese instant messaging programme) in reducing anaemia prevalence (trial registered through the China Ethics Committee for Registering Clinical Trials, registration number ChiECRCT-2012033). The trial took place in 7 out of the 16 townships in Zhao County (Hancun, Yanghu, Beizhongma, Beiwangli, Xinzhaidian, Gedatou and Daifuzhuang). These townships cover mainly rural areas of Zhao County, and have 107 villages with an estimated total population of 206,600, under-five population of 12,700 and 3600 children aged 6–23 months [33].

The sample included caregivers (mother, fathers, grandmothers, grandfathers and others) of children aged 6–23 months and were not selected based on their mobile phone usage. Caregivers were excluded if they had a child of a different age, if they were not willing to participate, or if they were unable to read or understand the informed consent materials.

There was no specific sample size calculation for the survey, because it was part of the trial. The sample size calculation for this trial was based on assumptions that will be reported elsewhere. It was anticipated that 2400 caregivers out of 3600 children on the list of names (70%) could participate and complete the survey (WW, personal communication).

**Semi-structured interviews.** The interviews took place in Zhaozhou Township (a township that was not included in the survey). Zhaozhou Township has the largest population (estimated total population of 109,200) of townships in Zhao County. Caregivers were recruited in Zhaozhou downtown area, which represents a semi-urban area in Zhaozhou Township, and in a village in a rural area of Zhaozhou Township [33]. Participants were eligible if they were a caregiver of young child and used a mobile phone. Caregivers who did not use a mobile phone were excluded, because these caregivers could not provide insights into the research questions related to factors influencing mobile phone usage. The sample was purposefully selected based on characteristics that were considered to be relevant: type of caregiver, age, urban or rural residence, education and type of mobile phone (simple mobile phone or smartphone).

The sample size had to be large enough to cover the diverse views of caregivers and to reach saturation of themes. It was planned to interview between 15 and 20 caregivers [34–36].

### Interviewers

**Survey.** Trained medical students from local universities conducted the survey and were guided by three supervisors (WW, YL and BL), who were all experienced in supervising surveys in Zhao County.

**Semi-structured interviews.** The interviewer (YL) conducted the interviews with caregivers in Mandarin and an observer (MV) was present during the interviews to assist the interviewer and to record any non-verbal communication and observations. The experiences and knowledge of the interviewer and observer complemented each other. The interviewer was a female native Chinese MSc student with a BSc in medical sciences and who grew up in Beijing. She was very familiar with the study context as she was involved in several studies that took place in Zhao County. The observer was a female PhD student with professional proficiency in the English language and had experience with qualitative interviews. She spent time in China and in Zhao County, but had few preconceptions about the study context. The researchers worked closely together to ensure validity of the findings.

### Recruitment

**Survey.** Recruitment of caregivers took place by asking them from “door-to-door” on the day and invite willing caregivers to come to the village clinic, where interviewers recruited them. Village doctors were asked to help with gathering caregivers in the village clinics, because many caregivers were familiar with their village doctor and more likely to participate when they were asked by their village doctor. Interviewers recruited caregivers and obtained informed consent from all participants. Participants were given a towel (worth ¥5 (approximately£0.52, €0.62, US$0.82)) for their time, which was also done in previous studies in Zhao County [25,37].

**Semi-structured interviews.** Village doctors were asked to find caregivers who were willing to participate. The interviewer did not know the caregivers prior to the interviews. The interviewer approached caregivers and asked them face-to-face if they were interested in participation. A snowballing method was used; caregivers were asked if they knew any other caregivers who were willing to participate. Participants were given a towel (worth ¥5) for their time.

### Questionnaires

Factors influencing usage of mobile phones known from the literature and factors based on our previous experiences were used to develop the questionnaires that are presented in S1 Appendix [5,11–13]. The questionnaires were developed at the same time and the questions were partly matched so that the results from the semi-structured interviews could provide more in-depth insights in the survey results.

**Survey.** Mobile phone usage related questions were developed by the research team and pilot tested with caregivers [26]. Demographic questionswere selected from the World Health Organization Maternal, Newborn and Child Health Household survey (unpublished, 2009). Questions were adapted to the local context in Zhao County and had been used in previous research [25].

**Semi-structured interviews.** The research team developed the interview guide and pilot tested the questions with caregivers [26]. Probing questions (asking open-ended questions; questions starting with how, why, what etc.) were used to follow-up on the questions in the guide, because an in-depth understanding of topics usually comes from probing [38].

### Data collection

**Survey.** Interviewers used a smartphone to record answers of caregivers in a private room in the village clinic, which was previously validated in the study setting[25].

**Semi-structured interviews.** The interviews were carried out at a neutral and private location that was comfortable for caregivers. This was often the caregivers’ home, or if that was not possible, a quiet room in the village clinic. The interviewer introduced the observer to the caregivers and explained the purpose of the observer’s presence. When the participant gave permission, the interview was recorded with a digital recorder, notes were taken to record non-verbal communication and photographs were taken of the caregiver and child (with face not identifiable and with their verbal and with written permission). The interviews took between 15 and 60 minutes.

The interviewer summarized her understanding of what the caregiver said twice during the interview to verify her understanding of the caregiver’s views. Because of this procedure, transcripts were not sent to caregivers. The interviewer translated parts of the interview content several times during the interview to allow the observer to ask additional questions. The researchers reflected after each interview and at the end of each field work day and recorded their ideas.

### Participants

**Survey.** A total of 1892 caregivers were surveyed and 1854 caregivers were included. The remaining 1708 caregivers of children (3600 children on the list of names minus 1892) could not be recruited, because they were not present in the village, the list of names was incorrect, or they did not meet the inclusion criteria. The following caregivers were excluded: 14 caregivers with children who were younger than six months or older than 23 months (the list of names could not be validated and there was a small time difference in time between obtaining the list of names and conducting the survey) and 24 records of children who shared a caregiver (caregiver had twins or two children aged 6–23 months and only the youngest child was included). Table 1 shows that of the 1854 included participants, most were mothers (1548; 83.5%), some were grandparents (260; 14.0%), and only a small proportion were fathers (41; 2.2%) or other caregivers (5; 0.3%). Table 2 shows the median ages, education and occupation of survey participants. Only few mothers (7; 0.5%) and no fathers (0; 0.0%) were not educated. Of the 176 grandparents who were primary caregivers of the children, more than a third (64; 36.4%) did not have education.

**Table 1:** Characteristics of survey participants and children (N = 1854).

**Gender of the child, n (%)**	
Boy	964 (52.0)
Girl	890 (48.0)
**Age of the child, n (%)**	
0–11 months	584 (31.5)
12–23 months	1270 (68.5)
**Number of children in the household, n (%)**	
1	946 (51.0)
2	833 (44.9)
3	71 (3.9)
4	2 (0.1)
Do not know	2 (0.1)
**Relation of participant to the child, n (%)**	
Mother	1548 (83.5)
Father	41 (2.2)
Grandmother or grandfather	260 (14.0)
Other caregiver^a^	5 (0.3)
**Participant is primary caregiver, n (%)**	
Yes	1722 (92.9)
No	132 (7.1)
**Additional other household member taking care of the child^b^, n (%)**	
Yes	296 (16.1)
No	1537 (83.8)
Do not know	2 (0.1)
**Registered as rural or urban, n (%)**	
Rural	1841 (99.3)
Urban	13 (0.7)
**Family net income in last year (¥)** ^c^	17,250 (20,000–30,000)
**Family living expenses in the last year (¥)** ^d^	15,000 (10,000–20,000)

^a^Child’s sister (1), child’s older brother’s wife (1), child’s father’s older sister (3).

^b^Two mothers discontinued the interview and did not answer this question and there were 17 missing values.

^c^1188 “Do not know”

^d^1129 “Do not know”

**Table 2:** Education and occupation of survey participants.

	Mothers(n = 1548)^a^	Fathers(n = 41)	Grand-parents(n = 176)^b^	Other caregivers(n = 5)^c^
**Age in years, median (Q1-Q3)**	26.0 (24.0–29.0)	28.0 (25.0–31.0)	51.0 (48.0–57.0)	30.0 (19.3–44.5)
**Education, n %**				
No education	7 (0.5)	0 (0.0)	64 (36.4)	0 (0.0)
Completed primary school (6 years of education, children start primary school from the age of six)	92 (5.9)	2 (4.9)	46 (26.1)	1 (20.0)
Completed junior high school (9 years of education)	1198 (77.4)	34 (82.9)	43 (24.4)	1 (20.0)
Completed senior high school (general education school, 12 years of education in total)	127 (8.2)	1 (2.4)	21 (11.9)	1 (20.0)
Completed secondary school (professional education school, 12 years of education in total)	68 (4.4)	2 (4.9)	1 (0.6)	0 (0.0)
Completed college (more than 15 years of education)	40 (2.6)	2 (4.9)	0 (0.0)	1 (20.0)
Completed university or above (more than 16 years of education)	5 (0.3)	0 (0.0)	0 (0.0)	0 (0.0)
Do not know	11 (0.7)	0 (0.0)	1 (0.6)	1 (0.0)
**Occupation,n %**				
Home	1386 (89.5)	0 (0.0)	158 (89.8)	2 (40.0)
Work	156 (10.1)	41 (100.0)	17 (9.6)	2 (40.0)
Do not know	6 (0.4)	0 (0.0)	1 (0.6)	1 (20.0)

^a^6 “do not know”.

^b^For grandparents these questions were only asked to primary caregivers, 1 “do not know”.

^c^1 “do not know”.

**Semi-structured interviews.** A total of 23 caregivers were approached and six caregivers were unable or refused to participate in the interview. The 17 interviewed caregivers included 13 parents (12 mothers and one father) and four grandparents, (two grandmothers and two grandfathers) (Table 3). Parents were aged between 24 and 33 years and grandparents were aged between 48 and 57 years. More than half (10/17) of participants used a smartphone.

**Table 3:** Characteristics of semi-structured interview participants.

Nr	Caregiver	Age years	Education level^a^	Occupation	Age child (months)	Gender child	Urban or rural	Nr of children	Type of mobile phone
1	Mother	25	7	Housework	1	Boy	Rural	1	Simple
2	Mother	24	3	Housework	10	Girl	Rural	2	Smart
3	Grandmother	57	2	Head organisation	28	Girl	Urban	1	Simple
4	Grandfather	57	3	Construction	25	Girl	Rural	1	Simple
5	Mother	27	3	Other	34	Girl	Rural	1	Smart
6	Mother	24	3	Housework	18	Girl	Rural	1	Smart
7	Mother	30	5	Housework	53	Boy	Rural	2	Smart
8	Mother	32	3	Housework	64	Boy	Rural	2	Smart
9	Mother	30	7	Technical	25	Boy	Urban	1	Simple
10	Mother	28	3	Housework	5	Girl	Unclear	2	Simple
11	Mother	24	3	Housework	14	Boy	Rural	1	Smart
12	Mother	24	3	Commercial	3	Girl	Rural	1	Simple
13	Mother	25	3	Housework	26	Girl	Rural	1	Smart
14	Mother	24	5	Housework	21	Boy	Urban	1	Smart
15	Father	33	3	Commercial	40	Girl	Rural	2	Smart
16	Grandmother	48	3	Housework	9	Girl	Rural	1	Simple
17	Grandfather	52	4	Commercial	0	Boy	Rural	1	Smart

^a^Education level:

1 = no education;

2 = completed primary school (6 years of education);

3 = completed junior high school (9 years of education);

4 = completed senior high school (general education school, 12 years of education in total);

5 = completed secondary school (professional education school, 12 years of education in total);

6 = completed college (more than 15 years of education);

7 = completed university or above (more than 16 years of education).

### Data management and analysis of outcomes

The survey and semi-structured interviews were analysed separately and the results were compared and integrated. The structure of the themes that were found in the semi-structured interviews was used for reporting; the survey results were added to these themes. We anonymised participant identifiable information for data analysis and reporting.

**Survey.** Data were wirelessly and securely uploaded into an Excel database via an Internet server. Data were also saved on the memory card of the smartphone as an encrypted file. Data could only be decrypted with special software. Only the supervisors were able to enter the databases and no changes could be made to the databases. SPSS version 16.0 [39]and SAS version 9.2 [40] were used for the analysis. A simple descriptive analysis was used to calculate proportions, medians (Q2), 25 (Q1) and 75 (Q3) percentiles for the demographic and mobile phone usagerelated variables [26]. Data were analysed for different groups of caregivers (mother, fathers, grandmothers, grandfathers and others), because their mobile phone usage was anticipated to be different. Missing data were not imputed.

**Semi-structured interviews.** The recorded data and transcripts were kept on a secure computer. A local student transcribed the recorded data verbatim in Word 2007. These transcriptions were checked by another student and second-checked by the interviewer by listening to the tapes. Then the interviewer translated the interviews into English entirely, which was checked by a second Chinese researcher (WW). Where there were discrepancies, WW and YL discussed the meaning of the transcripts. A bilingual translator checked a random selection of 10% of the transcripts to validate the translation. The transcripts were transferred in computer-aided qualitative data analysis software MAXQDA 11 [41]. To maximize rigour, the interviewer and observer conducted the analysis independently: the interviewer in Mandarin and the observer in English. The influence of the researchers’ social positioning was considered when analysing the data; meaning that the interviewer was seen by the interview participants as a young female medical researcher from Beijing and observer as a young female foreigner. From the openness of participants and richness of the data, it became clear that participants felt comfortable talking to the interviewer in presence of the observer.

A thematic analysis was used, because this is especially useful for under-researched areas. It was aimed to provide a rich thematic description of the entire data set that reflected the most important findings and it was anticipated that thorough insights in specific issues could not be obtained [42]. It was aimed to gain insights from dissonant cases; people who were unusual in some way.

Thematic analysis was conducted in six steps as described by Braun and Clarke[42]. Firstly, the researchers read through the interviews several times in an active way (searching for meaning) to obtain an overview of the interviews. They kept memos to capture thought processes. Secondly, initial codes were given to findings (units of texts). Thirdly, the researchers searched for themes and sorted codes into potential themes. They carried out this process independently and discussed and compared their findings. In the fourth step, the themes were reviewed and compared on two levels: of the coded data extracts and in relation to the data set. This was continued until a good idea was found of what the themes were and how they fitted in the data set. In the fifth step, themes were defined and named [42]. The interviewer translated the Mandarin themes into English and the observer compared these with the English themes. To validate the translation of the themes, the bilingual translator translated the final English version of the themes back into Mandarin and the interviewer compared these with the original Mandarin themes [43]. In the sixth step, different themes were related to each other to develop an explanation in relation to the research question. Vivid quotes which captured the essence of key points were chosen and the “story” was written[42]. To verify the data, field work memos and observations were compared with the analysed data. Moreover, the results were discussed within the research team to verify the understanding of the interpretation.

Both the English translations and the original Mandarin transcripts are provided for the themes and quotes. The interviewee related to the quotes and analyses are indicated as “I” followed by the number of the interviewee.

### Ethics statement

The Ethical Committee of the Capital Institute of Pediatrics in Beijing provided ethical approval (No. SHERLL 2013009). All participants provided written informed consent prior to their inclusion in the study. We anonymized participant identifiable information for data analysis and reporting. Permission was obtained from the local health officials at the Zhao County Health Bureau and Zhao County Maternal and Child Health Hospital. We obtained written consent by the subject for publication of the striking image.

## Results

We found four main themes: (i) trends in mobile phones ownership; (ii) usage of mobile phone functions; (iii) factors influencing replying to text messages; and (iv) uses of mobile phones for health care (Table 4).

**Table 4:** Overview of themes related to usage of mobile phones.

Themes			Subthemes	
Nr	Mandarin	English	Mandarin	English
1	手机所有权的趋势	Trends in mobile phone ownership	手机和智能手机	Mobile phones and smartphones
			使用手机多久了	Duration of mobile phone usage
			用手机的原因	Reasons for using mobile phones
			共用手机	Shared mobile phones
			获得手机方式	Method of mobile phone acquisition
			换手机	Changing mobile phones
2	手机的使用	Usage of mobile phone functions	手机使用中的问题	Problems whilst using mobile phones
			手机话费	Mobile phone bills
			短信	Text messages
			打电话	Phone calls
			发短信和打电话的比较	Comparison between making phone calls and sending text messages
			其他功能	Other functionalities
3	回短信的影响因素	Factors influencing replying to text messages	查看手机	Checking mobile phones
			信任发送者	Trusting the sender
			接到短信时心情	Emotion/feeling when receiving a text message
			回短信的重要性	The importance of replying to text messages
			短信的易用性	Ease of use of text messages
4	手机在卫生保健方面的应用	Uses of mobile phones for health care	给医生打电话	Calling the doctor
			给家人打电话	Calling family
			健康相关短信	Health-related text messages
			上网查询健康相关信息	Browsing the Internet for health-related information
			健康相关应用	Health-related apps

### Theme 1: Trends in mobile phone ownership

The first theme had the following six subthemes: (i) mobile phones and smartphones; (ii) duration of mobile phone usage; (iii) reasons for using mobile phones; (iv) shared mobile phones; (v) method of mobile phone acquisition; and (vi) changing mobile phones and SIM cards.

**Mobile phones and smartphones.** Table 5 shows that a large proportion of the 1854 survey participants (1620; 87.4%) used mobile phones. A considerably higher proportion of mothers (1433; 92.6%) and fathers (41; 100.0%) used mobile phones compared to grandparents (142; 54.6%). These findings were similar to when survey participants were asked about mobile phone usage of their household members (S1 Table).Table 5 shows that almost a third of the 1620 mobile phone using participants had a smartphone (487; 30.1%), but they were almost all mothers and fathers (484; 99.4%). However, about 1 in 10 caregivers did not know whether they used a simple mobile phone or smartphone (155; 9.8%).

**Table 5:** Mobile phone ownership of survey participants.

	All		Mothers		Fathers		Grandparents		Other caregivers	
	n (%)	N	n (%)	N	n (%)	N	n (%)	N	n (%)	N
**Using mobile phone**										
Yes	1620 (87.4)	1854	1433 (92.6)	1548	41 (100.0)	41	142 (54.6)	260	4 (80.0)	5
No	234 (12.6)		115 (7.4)		0 (0.0)		118 (45.4)		1 (20.0)	
**Type of phone**										
Smartphone	487 (30.1)	1620	470 (32.8)	1433	14 (34.1)	41	2 (1.4)	142	1 (25.0)	4
Simple phone	978 (60.4)		844 (58.9)		23 (56.1)		109 (76.8)		2 (50.0)	
Unknown	155 (9.6)		119 (8.3)		4 (9.8)		31 (21.8)		1 (25.0)	
**Number of mobile phones in household** ^a^										
>1	1806 (97.4)	1853	-	-	-	-	-	-	-	-
1	22 (1.2)		-	-	-	-	-	-	-	-
0	17 (0.9)		-	-	-	-	-	-	-	-
Do not know	8 (0.5)		-	-	-	-	-	-	-	-
**Place where mobile phone was bought** ^a^										
County	926 (57.2)	1619	832 (58.1)	1432	23 (56.1)	41	68 (47.9)	142	3 (75.0)	4
Village	269 (16.6)		246 (17.2)		5 (12.2)		18 (12.7)		0 (0.0)	
Town	158 (9.8)		136 (9.5)		7 (17.1)		15 (10.6)		0 (0.0)	
City	147 (9.1)		131 (9.1)		5 (12.2)		10 (7.0)		1 (25.0)	
Other^b^	39 (2.4)		33 (2.3)		0 (0.0)		6 (4.2)		0 (0.0)	
Do not know	80 (4.9)		54 (3.8)		1 (2.4)		25 (17.6)		0 (0.0)	
**Times mobile phone number was changed in last year** ^a^										
Never	1347 (83.2)	1619	1179 (82.3)	1432	38 (92.7)	41	128 (90.1)	142	2 (50.0)	4
Once	193 (11.9)		179 (12.5)		2 (4.9)		10 (7.0)		2 (50.0)	
Twice	49 (3.0)		47 (3.3)		1 (2.4)		1 (0.7)		0 (0.0)	
Three times	13 (0.8)		13 (0.9)		0 (0.0)		0 (0.0)		0 (0.0)	
Four times or more	8 (0.5)		7 (0.5)		0 (0.0)		1 (0.7)		0 (0.0)	
Unknown	9 (0.6)		7 (0.5)		0 (0.0)		2 (1.5)		0 (0.0)	

^a^One mother discontinued the interview and did not answer this question.

^b^Other ways to obtain a mobile phone were as follows: mobile phone was given by a shop when connecting to the Internet for their own computer or when they changing the mobile phone card (15); old mobile phone was received from another person (12); mobile phone was bought somewhere outside Zhao County (10); mobile phone was shared with others (2).

Many semi-structured interview participants had a smartphone (I2, 5–8, 11, 13–15, 17), but not everyone could use the more advanced functions of a smartphone (I7), or was aware of the smartphone operating system (I6). A phone that could be used to make phone calls was sufficient (I2).

Grandfather: “It is an information age, and our country has developed very fast. [Everybody] needs a mobile phone, though we don’t need a mobile phone with many functions”. “信息时代嘛，国内发展也不慢。必须有个手机，但是不需要多夸大”。(I17)

**Duration of mobile phone usage.** Mobile phones were commonly used for approximately 5 to 10 years (I1, 3, 6, 9, 10, 11, 13, 14), and sometimes less than 5 years (I2, 7, 12, 16) or more than 10 years (I4, 14, 15). Because the mobile phone has been widely used for a long time, caregivers were generally familiar with their usage (I8).

Mother: “…. Now who is not familiar with mobile phone use”? “现在谁使手机不熟悉啊”？ (I8)

**Reasons for using mobile phones.** Before mobile phones became widespread, landline telephones were used by some in Zhao County. The mobile phone replaced the landline telephone (I3–5, 16). Another reason for starting to use a mobile phone was because everyone had one (I6, 11–13, 17). Many found it convenient to contact others by a mobile phone (I1–12, 14, 17).

Grandfather: “[Everybody] in the family needs to have a mobile phone now”. “现在吧家人都有…都得有了”。

YL: “Why? Why does [everybody] need to have a mobile phone now”? “为什么都得有啊”？

Grandfather: “Because it is more convenient to get information by mobile phone and we can find [each other] easily. We had to go to other people’s homes before. Now a phone call is sufficient”. “就是信息方便呗，找找你吧，一找就到了，当时原先找你吧……还敲你门嘞或者什么的，现在打个电话就行了”。

YL: “Ok”. “恩”。(I17)

A mobile phone facilitated solving common daily life problems, such as informing someone when running late, asking someone to pick up the child from school, or asking for help when a bicycle had a flat tire (I7). Starting to work was a reason to use a mobile phone (I5, 11, 13). Also caregivers needed their mobile phones for work (I1, 3–5, 7, 9); for example to contact colleagues or customers (I1, 9). Mobile phones had become part of caregivers’ daily lives and they had formed a habit of using them (I10, 13).

**Shared mobile phones.** Table 5 shows that almost all households of survey participants had more than one mobile phone (1806; 97.4%). Of caregivers and their household members who used a mobile phone, almost all mothers (1653; 99.2%), fathers (1731; 98.5%), grandmothers (712; 95.8%) and grandfathers (850; 97.0%) owned a personal mobile phone (S1 Table).

In addition, the semi-structured findings showed that it was uncommon to share mobile phones permanently with others. Both caregivers and their family members usually had their own mobile phone. However, the interviews revealed that mobile phones were sometimes shared for a short period of time. It was commonly the father who used the mother’s mobile phone (I1, 2, 7).

**Method of mobile phone acquisition.** Table 5 shows that survey participants who used a mobile phone bought mobile phones most frequently in a county level shop (926; 57.2%) or in the local village (269; 16.6%).

Semi-structured interview findings also showed that mobile phones were usually bought in local shops. When a mobile phone was required for work, it was usually provided by the employer (I1, 4, 9). Mobile phones were also received as a gift from a relative. These gift mobile phones were either new (I6, 8, 14) and sometimes given for a special event such as a birthday (I6), or old and given when a relative bought a new mobile phone (I9, 16). Old mobile phones did not always function well, but this was not a problem when it was usable (I9).

Mother: “I think it was from a relative. The former mobile phone was broken. I could not use it and someone said he/she had an old one, so I used it. It is ok as long as it can make phone calls, because I do not use other functions”. “亲戚的吧，我上边那个手机是坏了，不能用了，然后别人说有一个旧的我就用了只要能打电话就行了，因为不用其他功能”。(I9)

**Changing mobile phones and SIM cards.** Table 5 shows that most survey participants who used a mobile phone did not change their mobile phone number in the past year (1347; 83.2%), and a relatively low proportion had changed it once (193; 11.9%).

Similar to these survey findings, also semi-structured interviews showed that caregivers did not change their mobile phone very often; it was common to change the phone approximately every two years, which was done when there were problems, or when it was disliked (I10). Sometimes then also the SIM card was changed, because it was convenient to buy both at the same time (I4). A high charge by the mobile telecom operator was another reason for changing SIM cards (I9).

### Theme 2: Usage of mobile phone functions

The second theme had the following six subthemes: (i) problems whilst using mobile phones; (ii) mobile phone bills; (iii) text messages; (iv) phone calls; (v) comparison between making phone calls and sending text messaging; and (vi) other functionalities.

**Problems whilst using mobile phones.** Table 6 shows that nearly all survey participants who used a mobile phone said that it functioned for calling and text messaging (1600; 98.8%).

**Table 6:** Usage of mobile phone for calls and text messages by survey participants, n (%).

	All (N = 1620)	Mothers (n = 1433)	Fathers (n = 41)	Grandparents (n = 142)	Other caregivers (n = 4)
**Functioning mobile phone** ^a^					
Functions for calls and text messages	1600 (98.8)	1422 (99.3)	41 (100.0)	133 (93.7)	4 (100.0)
Does not function for text messages	10 (0.6)	1 (0.1)	0 (0.0)	9 (6.3)	0 (0.0)
Does not function for calls	1 (0.1)	1 (0.1)	0 (0.0)	0 (0.0)	0 (0.0)
Does not function for calls and text messages	7 (0.4)	7 (0.4)	0 (0.0)	0 (0.0)	0 (0.0)
Other	1 (0.1)	1 (0.1)	0 (0.0)	0 (0.0)	0 (0.0)
**Primary usage of mobile phone calling versus texting**					
Calling	1475 (91.0)	1297 (90.5)	39 (95.1)	135 (95.1)	4 (100.0)
Text messaging	33 (2.0)	33 (2.3)	0 (0.0)	0 (0.0)	0 (0.0)
Both in equal measure	106 (6.5)	100 (7.0)	2 (4.9)	4 (2.8)	0 (0.0)
Other (Internet (2), QQ (1), do not know (3)	6 (0.5)	3 (0.2)	0 (0.0)	3 (2.1)	0 (0.0)
**Primary usage of text messaging function**					
Text messaging	631 (39.0)	579 (40.4)	25 (61.0)	26 (18.3)	1 (25.0)
QQ	540 (33.3)	532 (37.1)	6 (14.6)	1 (0.7)	1 (25.0)
Both in equal measure	198 (12.2)	184 (12.8)	7 (17.1)	5 (3.5)	2 (50.0)
Cannot use either	245 (15.1)	132 (9.2)	3 (7.3)	110 (77.5)	0 (0.0)
**Location where mobile phone is placed when leaving the house**					
Carry phone	1115 (68.8)	975 (68.0)	40 (97.6)	96 (67.6)	4 (100.0)
Leave phone in house	498 (30.7)	451 (31.5)	1 (2.4)	46 (32.4)	0 (0.0)
Someone else carries phone	5 (0.3)	5 (0.3)	0 (0.0)	0 (0.0)	0 (0.0)
Other (no fixed location, normally do not carry it)	2 (0.1)	2 (0.2)	0 (0.0)	0 (0.0)	0 (0.0)

^a^One mother discontinued the interview and did not answer this question.

In the semi-structured interview it was found that while some caregivers had no problems with their mobile phones (I3, 7), problems that limited calling and text messaging with a mobile phone were quite common (I4). S1 Text describes the following problems that occurred whilst using mobile phones: (i) non-functioning mobile phone; (ii) phone running out of credit; (iii) empty battery; (iv) lost mobile phone; and (v) radiation.

**Mobile phone bills.** Table 7 shows that survey participants spent a median of ¥20 (approximately £2.0, €2.4, US$3.2) (¥20–30) per month on their mobile phone.

**Table 7:** Number of calls and text messages used and amount of money spend by survey participants.

	All (N = 1620)	Mothers (n = 1433)	Fathers (n = 41)	Grandparents (n = 142)	Other caregivers (n = 4)
**Calls made per week**					
Number,median (Q1-Q3)	7 (4–10)	7 (4–10)	7 (4–25)	5 (2–7)	6 (6–6)
Answered question, n (%)	880 (54.3)	790 (55.1)	19 (46.3)	70 (49.3)	1 (25.0)
Do not know, n (%)	725 (44.8)	633 (44.2)	21 (51.2)	68 (47.9)	3 (75.0)
Unable to make call, n (%)	15 (0.9)	10 (0.7)	1 (2.5)	4 (2.8)	0 (0.0)
**Calls received per week**					
Number,median (Q1-Q3)	7 (4–10)	7 (4–10)	10 (6–26)	7 (5–8)	6 (6–6)
Answered question	847 (52.2)	758 (52.9)	20 (48.8)	68 (47.9)	1 (25.0)
Do not know	764 (47.2)	669 (46.7)	20 (48.8)	72 (50.7)	3 (75.0)
Unable to receive call	9 (0.6)	6 (0.4)	1 (2.4)	2 (1.4)	0 (0.0)
**Text messages sent per week**					
Number,median (Q1-Q3)	0 (0–4)	0 (0–5)	0 (0–4)	0 (0–0)	-
Answered question	960 (59.3)	857 (59.8)	27 (65.9)	76 (53.5)	0 (0.0)
Do not know	540 (33.3)	491 (34.3)	14 (34.1)	31 (21.8)	4 (100.0)
Unable to send text message	120 (7.4)	85 (5.9)	0 (0.0)	35 (24.7)	0 (0.0)
**Text messages received per week**					
Number,median (Q1-Q3)	7 (2–10)	7 (2–10)	5 (2–7)	4 (0–7)	-
Answered question	931 (57.5)	835 (58.3)	24 (58.5)	72 (50.7)	0 (0.0)
Do not know	641 (39.6)	575 (40.1)	17 (41.5)	45 (31.7)	4 (100.0)
Unable to receive text message	48 (2.9)	23 (1.6)	0 (0.0)	25 (17.6)	0 (0.0)
**Phone bill per month (¥)**					
Amount,median (Q1-Q3)	20 (20–30)	20 (20–30)	30 (25–55)	20 (10–25)	35 (21–124)
Answered question	1443 (89.1)	1293 (90.2)	37 (90.2)	109 (76.8)	4 (100.0)
Do not know	177 (10.9)	140 (9.8)	4 (9.8)	33 (23.2)	(0.0)

Semi-structured participants mentioned to spent similar amounts of money on their mobile phones. These costs were seen as cheap (I2, 6, 7, 9).

**Text messages.** Table 7shows that the median number of text messages sent was 0 per week (Q1-Q3; 0–4) and the median number of text messages received was 7 per week (Q1-Q3; 2–10). Of 1433 surveyed mothers and 41 surveyed fathers, few mothers (120; 7.4%) and fathers (85; 5.9%) were unable to send text messages. However, of 142 surveyed grandparents only about one in four were unable to send text messages (35; 24.7%). S1 Table shows that when survey participants were asked the ability of their household members to text message these proportions were similar for parents, but a much higher proportion of grandparents were thought to be unable to send text messages (76.4% of grandfathers and 68.6% of grandmothers).

Semi-structured interviews participants also did not usually sent text messages and mentioned more frequently receiving than sending text messages (I3, 7). Parents used text messaging more often than grandparents. Grandparents had much more difficulty with text messaging or could not use this function at all (I3, 4, 16, 17). Grandparents could still read text messages and sometimes asked others for help with sending text messages (I4). One grandfather could only type very short text messages (I17). A grandmother could use text messages with her previous mobile phone, but had not learnt it on her new mobile phone. Not remembering pinyin (pinyin is the official phonetic system for transcribing the Chinese pronunciation of Chinese characters into the Latin alphabet) well was another problem (I4, 17). As making phone calls was sufficient, there was no need to learn to use text messages (I4).

Grandmother: “Eh, because after I changed a new phone, there is no need to learn that. I did not read that. At our age, it has no use. I do not send text messages and so on, and just making and receiving calls is enough. The.. I did not need it for other things. Haha… .. ” “呃…换手机后来没什么事了我都不看那，光着…咱这岁数了也没用，也不发短信也不干嘛，光能接能打就成了，就是那个…不要求说说干什么别的是吧（呵呵）那……” (I16)

Some parents disliked sending text messages, because they were not used to it (I5, I15). Text messaging was perceived to be something that younger people liked to do (I10). However, some liked to commonly send text messages (I9). Text messaging was particularly useful for simple things and when there was nothing urgent (I5, 10, 12). Text messages were mainly sent to friends, classmates, colleagues and sometimes to family members (I1, 2, 5, 6, 9–11).

Some never received text messages from people they knew (I14, 16). Receiving text messages that were perceived as “junk” or “scam” were quite common (I11–13, 15, 17) and included text messages from mobile telecom operators. Caregivers liked to receive useful text messages such as reminder messages about running out of mobile phone credit, because they were informative (I15). Some subscribed to a text messaging service that provided the news or weather information (I1). It was also popular to receive text messages for fun, such as jokes, or blessings during Chinese festivals (I11) that were forwarded to others (I8).

**Phone calls.** Table 7shows that the median number of calls made was 7 per week (Q1-Q3; 4–10) and this number was the same for the number of calls received per week. Of 1433 surveyed mothers, 41 surveyed fathers and 142 surveyed grandparents, very few mothers (15; 0.9%), fathers (1; 2.5%) and grandparents (4; 2.8%) were not able to make a phone call. S1 Table shows that these proportions were similar for all caregivers when survey participants were asked the ability of their household members to make a phone call.

These survey findings were consistent with the semi-structured interview findings. All parents and grandparents were familiar with making phone calls. Some caregivers made phone calls frequently; often a number of calls on a day (I1, 2, 3, 9, 13, 14). Calls were made to family members when there was something urgent (5), to see how a family member was doing (I1, 6–8, 13, 14), to talk about daily things (I8, 10, 13) or to talk about the child (I1, 6, 9, 10). No calls were made later in the evening, because caregivers did not want to disturb others (I8). Grandparents often took care of their grandchild. They used the mobile phone to let the child and parents talk to each other when parents were away (I3).

Grandmother: “About something new that the child learned. (For example) the poetry of Tang Dynasty the child has learned, or the songs and the children’s songs and the ballad she has learned. And I will make a call to let the child sing to her parents. That’s all”. “就是孩子学什么新本事了，学会唐诗了，学会唱歌了，学会说儿歌了，说歌谣了，打个电话唱歌叫他妈妈爸爸听听，就是这”。(I3)

**Comparison between phone calls and sending text messages.** Table 6 shows that the mobile phone’s primary usage was calling for a high proportion of participants (1475; 91.0%). Only a very small proportion of participants mentioned text messaging as primary usage (33; 2.0%) and they were all mothers. Few participants mentioned to use both calling and text messaging in equal measure (106; 6.5%). When participants were asked about their preference for text messaging or QQ, more than a third of the 1620 participants preferred text messaging (631; 39.0%) and a third preferred QQ (540; 33.3%). About 1 in 10 participants used text messaging and QQ in equal measure (198; 12.2%). A relatively high proportion of grandparents could not use either text messaging or QQ (110; 77.5%) compared to mothers (132; 9.2%) and fathers (3; 7.3%).

These survey findings were consistent with semi-structured interviews. All grandparents and most parents preferred making phone calls, though some said to prefer text messaging (I5, 9). In S2 Text, the following factors related to preferences are described: (i) immediacy; (ii) clarity; (iii) ease of use; and (iv) costs.

**Other functionalities.** Only one survey participant mentioned Internet and one mentioned QQ as primary function of the mobile phone.

Semi-structured interview participants mentioned that other mobile phone functions they used included Internet (I8, 10–12), photos (I7, 9, 17), videos (I7, 9), recording sounds (I9), alarm (I1) and playing games (I10, 13). QQ was frequently used to chat with friends or colleagues, or to play games (I11, 13–15).

### Theme 3: Factors influencing replying to text messages

The third themehad the following five subthemes: (i) checking mobile phones; (ii) trusting the sender; (iii) emotion/feeling when receiving a text message; (iv) the importance of replying to text messages; and (v) ease of use of text messages.

**Checking mobile phones.** The frequency of checking mobile phones was variable. Some always checked their mobile phone (I13–15), while others usually did not check their mobile phone often (I10). When caregivers checked their mobile phone depended on the circumstances (I6, 13, 17), but caregivers said they generally responded when seeing a missed call or text message (I6, 10, 11). In S3 Text, factors related to checking the mobile phone are described: (i) when the user is free; (ii) where the mobile phone is placed; (iii) mobile phone switched on; and (iv) mobile phone audio volume.

**Trusting the sender.** Trust was related to who sent a text message. There were many text messages that were perceived as “scam” (I12, 15). Some of those text messages could easily be identified (I10).

Mother: “Yes. Or I can know it is a scamming message at a glance. Then I will not respond to it”. “恩，一看就是那诈骗短信那样的，肯定就不回”。(I10)

Caregivers feared having to pay a lot for calls or text messages of which they thought that were scamming (I11, 17). Text messages about selling various goods or about sending money were frequently received and perceived as harassing (I12, 15, 17). Often these text messages were not read, because they were useless (I12, 15). When it could not be easily seen whether the information in a text message was reliable or not, sometimes verification was sought by contacting the person who sent the text message. Some caregivers only responded to calls and text messages from known numbers of relatives and friends (I10, 11, 13–15), because there was no reason to reply to messages from unknown numbers (I13).

**Emotion/feeling when receiving a text message**. Although only mentioned once, a finding was that when being in a good mood, it was more likely to respond to a text message (I5).

Father: “I don’t know how to say that. It just depends on my mood. I will reply when I am in a good mood”. 那也说不清嘞，看心情嘞，心情好嘞就给他回下”。 (I15)

**The importance of replying to text messages.** Some text messages did not require a response, for example, information text messages from a mobile telecom operator (I14). Text messages that contained a question needed a response (I12). When the content of a text message was perceived as important and urgent, a reply was sent quicker (I14).

YL: “Ok. Then when you receive a message, I mean,when you see a message,how long will it take for you to reply to a message? “哦，那收到短信之后，就是您看到短信之后您多长时间才能回短信啊”。

Mother: “It depends on the content of the message. If it is very important, I will reply immediately; if it is not very important, such as those messages sent from the China Mobile service centre. I will not reply to those messages. “那要看什么短信，如果是有事短信的话，立马就回。如果没事的话，有些不是那个那个移动台上有时候给你发短信，那些短信都不回”。(I14)

Text messages that were perceived as unimportant were often not even read, because it was expected that a phone call would be made for important urgent matters (I10).

**Ease of use of text messages.** Some caregivers were only able to read the text messages, but were not used to responding to them (I15). The length of the reply that was required influenced the amount of effort it was to reply (I17). As previously mentioned, some caregivers and particularly grandparents found it hard to write text messages or were not able to text message (I17).

Grandfather: “Oh, it is too hard for me to reply a message. I will reply immediately if it just needs me to type yes, ok, but I’m not sure if it needs me to type many characters. About ten minutes, twenty minutes or more. You see”. “哎呀，那个我得需要回一条短信啊我得费劲了，你要说你要说我来了，或者是什么嗯，是的，那个我能马上就回，但是你要我编，就那意思写点东西可就慢，不敢说，得十分八分的，二十分的，是吧”。(I17)

### Theme 4: Uses of mobile phones for health care

Table 8 shows that more than half of households of caregivers with a mobile phone had the phone number of a health facility (936; 57.8%). About one in four participants (432; 26.7%) had used their mobile phone in the past three months for health care. However, of the 1187 participants who had not used their mobile phone for health care, a very high proportion wished to use their phone to receive health information (1110; 93.5%).

**Table 8:** Usage of mobile phones for health care by survey participants.

	**All**		**Mothers**		**Fathers**		**Grandparents**		**Other caregivers**	
	**n (%)**	**N**	**n (%)**	**N**	**n (%)**	**N**	**n (%)**	**N**	**n (%)**	**N**
**Household having the phone number of a health facility**										
Yes	936 (57.8)	1619								
No	680 (42.0)									
Do not know	3 (0.2)									
**Household having the phone number of the county hospital or above**										
Yes	339 (20.9)	1619								
No	1275 (78.8)									
Do not know	5 (0.3)									
**Household having the phone number of the township hospital**										
Yes	344 (21.2)	1619								
No	1269 (78.4)									
Do not know	6 (0.4)									
**Household having the phone number of the village clinic**										
Yes	763 (47.1)	1619								
No	853 (52.7)									
Do not know	3 (0.2)									
**Used phone to receive health information in past 3 months** ^a^										
Never	1187 (73.3)	1619	1033 (72.1)	1432	26 (63.4)	41	124 (87.3)	142	4 (100.0)	4
1x	90 (5.6)		80 (5.6)		4 (9.8)		6 (4.2)		0 (0.0)	
2x	97 (6.0)		92 (6.4)		1 (2.4)		4 (2.8)		0 (0.0)	
3x	125 (7.7)		116 (8.1)		6 (14.6)		3 (2.1)		0 (0.0)	
>3x	120 (7.4)		111 (7.8)		4 (9.8)		5 (3.6)		0 (0.0)	
**Like to use mobile to receive health information**										
Yes	1110 (93.5)	1187	973 (94.2)	1033	25 (96.2)	26	108 (87.1)	124	4 (100.0)	4
No	73 (6.1)		57 (5.5)		1 (3.8)		15 (12.1)		0 (0.0)	
Other	4 (0.4)		3 (0.3)		0 (0.0)		1 (0.8)		0 (0.0)	

^a^One mother discontinued the interview and did not answer this question.

The following sections describe the findings of the semi-structured interviews, in which the following was mentioned: (i) calling the doctor; (ii) calling family; (iii) health-related text messages; (iv) browsing the Internet for health-related information; and (v) health-related apps.

**Calling the doctor.** It was common to call a doctor (I2–5, 7, 10, 13, 14); often the village doctor (I3–5, 7, 10, 14). Generally the village doctor was called once in a while, but sometimes up to two or three times a day when there was an issue (I4). Calling was used as an alternative way of reaching the village doctor when the doctor was out of the clinic (I4). It was found to be convenient to call a doctor, because it provided caregivers with direct contact without having to go out (I5, 7). Calls were used for advice about the child’s symptoms or medicines (I5, 7, 13, 14). Sometimes the doctor visited the participant after a mobile phone call (I4). The “immunization doctor” was called for advice about vaccinations (I2, 3, 13, 14).

Mother: “… . Last time there were drugs for flu or something else. He said it was very short of supply. Sometimes, when you get there, there are no drugs. You can make phone calls to him/her to see whether there is the supply of the drug. If there is, then you can bring your child to there, right? Sometimes, it turns out that he/she is not there. Your child might catch a cold from travelling there and to and fro too much”. “他这一管针可能说说扎好几个孩子的，都用这个药，比如说你去了以后没这个药，就是上次有一个什么好像是流感药还是什么药，他说特别紧缺哈。有时候你去就没有这个药比如你打电话问他一下说看你们现在这个药到了吧？到了就带孩子去扎嘞，是吧，有时候你去了他没在，跑来跑去怕孩子受凉”。(I2)

Some caregivers had never called a doctor (I1, 6, 11, 16, 17). Reasons for this varied and included not having thought about calling a doctor (17) and finding it unnecessary to call a doctor (I11, 16), because it was the role of the village doctor to be available at the clinic (I16). Also the husband of one mother worked in the hospital and he could easily ask health-related questions (I1). There was no use in calling when the village clinic had to be visited for medicines anyway. This was no problem, because it was easy to go when living near the village clinic (I11).

**Calling family.** Calls were made to family members when there was something wrong with the child (I9, 10). In addition, it was common for family members to call each other to check up on their health condition (I2, 3, 6, 8).

**Health-related text messages.** Text messages were not sent to ask for health advice, but text messages with health information were often received. Text messages from the immunization centre reminded caregivers to come to the centre for vaccination of their child (I10, 14, 15). Health information text messages commonly had general health advice and were sent by mobile telecom operators (I7, 10).

Mother: “Yes. I received some text messages from the operator (China Mobile^a^) telling me to drink more water or eat more pears^b^ during the winter or some other things”. “哦，那就是那个移动上边有时候发短信告诉那个多喝水啊，或者冬天里多吃梨啊，或者什么的嘛” 。(I7)

^a^China mobile is one of the main state-owned mobile telecom operators. This particular message was a health message.

^b^Zhao County is famous for its pears [44].

Other health text messages were about child feeding, development (length, use of left or right hand, crouching, standing, walking etc.) and prevention of colds by giving the advice to wear more clothes (I10, 13). These text messages were perceived as good, were read and the advice was followed (I10, 12). It was convenient that those messages did not need a reply (I13).

**Browsing the Internet for health-related information.** The Internet was used to search for health information. Sometimes the mobile phone was used (I8), but mainly the computer in the home was used (I12). When a condition was not so serious, the Internet was searched first. This reduced worries and gave a safe feeling (I8). Searching the Internet was perceived as convenient, because information about the child’s health could be found, for example about the child’s height (I11) and for acute problems, such as when the child had a cold (I12).

Mother: “If he just has a cold with a runny nose, medicine is not needed, while it is necessary if he has a fever with coughing [according to the website]”. “他就说是感冒似的，流鼻子不用吃药。如果是发烧咳嗽了吃点药” 。(I12)

Conversely, the information on the Internet was unclear and a search had to be done many times (I8). The information on the Internet seemed useless, because the information was not reliable (I12). Seeing a doctor was then preferred, because a doctor was trusted more than information on the Internet (I8, 12).

**Health-related apps.** Although half of caregivers used a smartphone, no one claimed to use health apps. The reason for this was unclear, but downloading an app may be a barrier (I13).

Mother: “[I] needed to download something… .what’s the name… .sorry, I forgot it. Anyway, I got nothing and I needed to download an application… . I think”. 还得下什么 ……那叫嘛啊 忘了什么了，也反正是查不出来……得下那个应用好像” 。(I13)

## Discussion

### Principal findings

We found four main themes in the qualitative semi-structured interview data, which we compared and complemented with the quantitative survey data.

First, a large proportion of caregivers in ZhaoCounty in rural China used mobile phones. However, considerably more parents used mobile phones compared to grandparents. High usage of mobile phones, especially among parents, can be a facilitator for research. Most caregivers and their household members owned a personal mobile phone and had not changed their mobile phone number in the past year. Infrequent sharing and changing mobile phones may allow researchers to reach caregivers confidentially and follow a majority of caregivers up on the same mobile phone number. However, we found that sometimes family members shared mobile phones temporarily and caution is needed for these cases, which could result in privacy issues for mHealth-based interventions.

Second, almost all caregivers had mobile phones that functioned for calling and text messaging at the time of the survey. Qualitative findings showed that caregivers occasionally experienced problems related to a non-functioning mobile phone, phone running out of credit, empty battery, lost mobile phone and radiation. Most caregivers used their mobile phones primarily for calling. Qualitative findings showed that caregivers preferred making phone calls over sending text messages because it was quicker, clearer to communicate, required less effort and was cheaper. Most surveyed mothers and fathers were able to send text messages. However, many grandparents were unable to send a text message. An important reason for this was not being familiar with the pinyin system that is used for typing Chinese characters on mobile phones.

Third, several factors influenced whether caregivers replied to text messages, including: whether they checked their mobile phone; trusted the sender of the text message; their personal emotion or feeling when receiving a text message; the importance of replying to a text message; and ease of use of sending text messages. Ease of use was influenced by the length of the text message response.

Fourth, there were several uses of mobile phones in health care. Almost half of households had the phone number of a village clinic and about one in four caregivers had used their mobile phone for health care during the three months prior to the survey. Of those had not used their mobile phone for health care, a very high proportion wished to use their mobile phone to receive health information. Qualitative findings showed that mobile phones were used to call doctors and family members, receive health-related text messages and browse the Internet for health information. Mobile phones were not used for health-related apps.

### Comparison with relevant research

The findings on mobile phone ownership and usage were consistent with previous observations in Zhao County [25,37] and with a mixed methods study in Chongqing Province in western China [24]. Qualitative findings in the study in western China revealed that mobile phones were “convenient”, “necessary” and “affordable” [24]; which was similar to our findings. Lower rates of mobile phone ownership were found in two Kenyan studies conducted in 2009 [22]and 2012 [23].

Our study found that some caregivers had received their mobile phones as a gift from a relative. A study in Hubei Province in Northern China conducted by Nokia Research provided a rich description of Chinese mobile phone gifting practices and the social relations underlying them[45].

Text messaging is found to be user-friendly, because of its immediacy, low costs and non-intrusiveness [16]. However, the limited capacity of text messages (restricted number of characters), language and illiteracy have been found as barriers to its usage [4]. In a Kenyan study, of the 1137 mobile phone users, 914 (80.4%) could receive text messages, while 227 (20.0%) were unable to use text messaging and never read them [23]. In Zhao County, literacy rates are high and most parents were able to text message, but many grandparents were not. This was also found in a text messaging data collection study in Australia [46]. A study in the United Kingdom found that older participants would benefit from extensive training to implement appointment and medication reminder text messages [47].

Previously reported barriers to the usage of mobile phoneson an individual level in low- and middle-income countries included costs, lack of electricity, network problems, sharing or borrowing a mobile phone and lost or stolen mobile phones [11,12]. In our study costs, illiteracy, lack of electricity and sharing mobile phones did not seem to be large barriers to mobile phone usage. However, network problems and lost mobile phones were more occasionally a problem.

In addition, caregivers expressed their concerns about radiation from mobile phones. The risks of mobile phone radiation has been a research area of interest, though definite conclusions have not been made [48]. Although this has been mentioned in the mHealth literature [49,50], no previous studies reported on the perception of participants in mHealth-based studies.

### Strengths and limitations

This study revealed insights in an underexplored research area. Use of mixed methods was beneficial, because quantitative and qualitative findings could be compared and complement each other. The survey provided quantitative data on mobile phone prevalence and usage, while the semi-structured interviews complemented these data by providing more in-depth insights. However, as a broad range of topics was explored, the results only provided a general overview of the research area of interest.

The semi-structured interview sample included a variety of caregivers from semi-urban and rural Zhao County, of different ages, levels of education and using simple mobile phones and smartphones. These findings can be transferable to settings with similar characteristics as Zhao County in China.

A limitation of the survey was that it was part of the baseline survey of the randomised controlled trial and thus did not have a probability sample. Although the survey may not necessarily be representative of Zhao County, almost all caregivers (99.3%) were from rural areas of Zhao County. Therefore, the survey can mainly be generalised to caregivers living in rural areas, though most people in Zhao County live in rural areas (90.7%).

Furthermore, external validity of the study may be limited by the design and implementation of the survey. Survey participants were interviewed in the village clinic rather than in their households, which may influence validity of some indictors. However, most indicators were not sensitive in the study setting and therefore this effect is likely to be minor. The study’s validity was also influenced by accuracy of the questions. The questions on demographic characteristics were taken from the World Health Organization Maternal Newborn and Child Health Household survey. The questions about income and expenses had a relatively high proportion of missing values, because participants did not know this information or did not want to give it. This is not surprising given that misreporting of income is frequent in low- and middle-income countries [51].

The questions on mobile phone usage questions were specifically designed for this survey. Although they were tested and piloted, some questions’ accuracy may be low. For example, survey participants often were unable to answer the questions about the number of mobile phone calls and text messages and thus these questions had high proportions of missing values (ranging from 33.3% to 44.7%). Participants said that their behaviour depended on whether something happened and that it was hard to give an estimation. Therefore, the answers to these questions about number of calls and text messages made per week were likely to be biased, but still can be used for a rough estimation.

Social desirability, language and interpretation bias were threats to the semi-structured interviews and were reduced as far as possible. First, the interviewer and particularly the observer’s presence as a foreigner could have introduced socially desirable answers. To reduce bias, the observer spent time in the local site and behaved in a culturally appropriate way. Also the interviewer who was very familiar with the study context introduced the observer to the participants and explained the purpose her presence. The richness of the data showed that the influence of the social position of the researchers was minimal.

Second, all efforts were made to reduce bias from language. The interviewer translated the findings, which were checked by another Chinese researcher and the observer. Moreover, the main findings were translated back by a bilingual translator.

Third, misinterpretation of the findings was minimized by strong collaboration between the interviewer and observer. They analysed the interviews separately. Also they spent a lot of time to compare each line of each interview, compare codes that were given and discuss their findings. Several drafts of the analysis were discussed within theresearch team to increase validity.

Finally, this study took place in January 2013 and changes in mobile phone use will likely have taken place between the study and publication of this paper. For example the instant messaging application “WeChat” is increasingly used throughout China on mobile phones [52]. While there are similarities between text messaging and messaging via a mobile application, the findings of this study regarding text messagingbehaviours cannot be directly translatedto instant messaging applications.

### Future research

mHealthresearchis often a “moving target”; this means that during the time that mHealth-based interventions are in the process of development and evaluation, people’s use of the technologies that can be used to assist delivery of interventions can change rapidly. Text messaging has been used over the past 20 years and is expected to remain to be the most important source for mobile messaging in many countries for some time to come [53]. Smartphones are increasingly available in low- and middle-income countries with rapidly decreasing prices of handsets. In the rural setting of this thesis, already approximately one in three parents owned a smartphone. Furthermore, it has been predicted that Internet connectivity will grow exponentially in the next 10 years and “everything, everywhere will be digitally connected” [54].

With these advancements, mobile instant messaging will increase its share in mobile messaging. Mobile instant messaging is forecast to increase from 1.6 trillion messages in 2011 to 7.7 trillion in 2016 [53]. Instant messaging can overcome the challenges presented by the limited capacity of text messages. Also, instant messages can be sent for free when a smartphone is connected to the internet.

However, even when smartphones are more widely used, that does not mean that people are comfortable with using them (as was reported in the results section of this paper). The growing complexity of technologies may also increase problems that are faced whilst using them. For example, at the moment smartphones have a much shorter battery life than simple mobile phones and instant messaging applications are dependent on Internet connectivity, which may not be readily available. In addition, people can sent and receive a large number of instantmessages in a short amount of time, which may reduce their attention forindividual messages. Thus, research needs to assess usage of smartphones and instant messaging by target populations before conducting intervention studies. Future studies could also explore issues more in-depth to improve user experiences and inform specific mHealth-based interventions.

### Conclusions

High mobile phone prevalence and usage that were found in this study can serve as facilitators for mHealth-based studies in Zhao County and settings with similar characteristics. The factors that were found to influence mobile phone usage can be used in these studies. In addition, the exploration of the natural role of mobile phones in health care can facilitate their integration in the health system. Future studies can explore issues in-depth to optimize experiences of users of mHealth-based interventions. In addition, as use of mobiles is changing, the use of new technologies, including smartphones and instant messaging, should be assessed.

## Supporting Information

S1 Appendix Questionnaires. (DOCX)

S1 Table Survey participants’ and household members' mobile phone usage (N = 1854). (DOCX)

S1 Text Theme 2: Usage of mobile phone functions; Problems whilst using mobile phones. (DOCX)

S2 Text Theme 2: Usage of mobile phone functions; Comparison between phone calls and sending text messages. (DOCX)

S3 Text Theme 3: Replying to text messages; Checking mobile phones. (DOCX)
