# Supplementary material for: Prevalence of Mobile Phones and Factors Influencing Usage by Caregivers of Young Children in Daily Life and for Health Care in Rural China: A Mixed Methods Study
Source: PLoS One. 2015 Mar 19;10(3):e0116216. doi: 10.1371/journal.pone.0116216 (PMC4366174; doi:10.1371/journal.pone.0116216)
Supplement: S1 Appendix — (DOCX) [file pone.0116216.s001.docx]

**Appendix S1**

**Questionnaires**

### Survey on demographics and mobile phone use - English version

| **Question nr** | **Question** | **Answer options** | **Referral to other questions** |
| --- | --- | --- | --- |
| **Identification (ID)** | | | |
| ID1a | The name (code) of the township | ________ |  |
| ID1b | The name (code) of the village | ________ |  |
| ID1c | The ID of the child | ________ |  |
| ID2 | The name (code) of the interviewee | ________ |  |
| ID3 | Relationship to the child | 1.Mother  2.Father 3.Grandfather/Grandmother 8.Other____ |  |
| ID4 | Who is the primary caregiver for this child? | 1.Mother  2.Father 3.Grandfather/Grandmother 8.Other____ |  |
| ID5 | The name of the child *(whose age is 6-23 months)* |  |  |
| ID6 | Gender of the child | 1.Male  2.Female |  |
| ID7 | Date of birth of the child *(solar calendar; yyyy/mm/dd)* | _______ |  |
| ID8 | Weight at birth | _______g  8888=Do not know |  |
| ID9 | Length at birth | _______cm  88=Do not know |  |
| ID10 | How many pregnancies has the mother of the child had, up to and including this child? | 8=Do not know |  |
| ID10a | How many children has the mother had before this child? *(fill in “7” if there are more than 7 children)* | __________ 8=Do not know |  |
| ID10b | Is the child a twin/triplet etc.? | 1.No  2.Twin  3.Triplet or more  8.Do not know |  |
| ID11 | What was the method of delivery for this child? | 1.Vaginal delivery  2.Delivery with instrumental assistance  3.Caesarean section  8.Do not know |  |
| ID12 | [Gestation period](app:ds:gestational) for this child in weeks *(round down to nearest week)* | ______________  88.Do not know |  |
| ID13 | Type of Hukou *(household register)* | 1.Urban  2.Rural |  |
| ID14 | Investigator ID | ____________ |  |
| ID15 | Date of the investigation *(solar calendar; yyyy/mm/dd)* | ____________ |  |

**Mobile phone (MP) use**

| MP1 | | Do you use a mobile phone? | | 1.Yes  2.No | If MP1=2 go to MP17 | |
| --- | --- | --- | --- | --- | --- | --- |
| MP2 | | What is the brand and type of the mobile phone you use? | | ___________________  8.Do not know |  | |
| MP3 | | Is the mobile phone you use a smartphone? *(demonstrate a smartphone, based on Android, Symbian or iOS system)* | | 1.Yes  2.No  8.Do not know |  | |
| MP4 | | What is the usual location of the mobile phone you use, when you leave the house? | | 1.Carry the phone with me  2.Leave the phone in the house  3.Someone else carries the phone  8.Other____ |  | |
| MP5 | | Do you primarily use a mobile phone for making calls or sending text messages? | | 1.Making calls  2.Sending text messages  3.Both in equal measure  8.Other____ |  | |
| MP5a | | Do you primarily send text messages or use QQ? | | 1.QQ  2.Text messages  3.Both in equal measure  4.Cannot use neither  5.Other____  8.Do not know |  | |
| MP6 | | How many phone calls do you make in an average week? | | __________________  88=Do not know  99=Cannot do it |  | |
| MP7 | | How many phone calls do you receive in an average week? | | __________________  88= Do not know  99=Cannot do it |  | |
| MP8 | | How many text messages do you sent in an average week? | | __________________  88=Do not know  99=Cannot do it |  | |
| MP9 | | How many text messages do you receive in an average week? | | __________________  88=Do not know  99=Cannot do it |  | |
| MP10 | | How much is your average mobile phone bill in one month? | | __________________  888=Do not know |  | |
| MP11 | | How often did you change your mobile phone number in the last year? | | 1.Never  2.Once  3.Twice  4.Three times  5.Four times or more  8.Do not remember |  | |
| MP12 | | Is your mobile phone currently functioning correctly i.e. can it be used for making a phone call and sending a text message? *(including a mobile phone without sufficient credit)* | | 1.The phone can be used to make a call and send a text message  2.The phone can be used to make a call but cannot send a text message  3.The phone can be used to send a text message, but cannot make a call  4.The phone can neither send a text message nor make a phone call  5.Other____ |  | |
| MP13 | | Do you or anyone in your household have the telephone number of the following *(including mobile number)*: | |  |  | |
|  | | 1.County hospital or above *(including county hospital, county children’s hospital, private hospital)* | | 1.Yes  2.No  8.Do not know |  | |
|  | | 2.Township hospital | | 1.Yes  2.No  8.Do not know |  | |
|  | | 3.Village clinic | | 1.Yes  2.No  8.Do not know |  | |
| MP14 | | How often have you used your mobile phone to receive information about your own health or your child’s health in the past 3 months? *(including text message and calls)* | | 1.Never  2.Once  3.Twice  4.Three times  5.More than three times | If MP14=1, go to MP15  If MP14=2,3,4 or 5, go to MP16 | |
| MP15 | | Would you like to use your mobile phone to receive health information? | | 1.Yes  2.No  3.Other____ |  | |
| MP16 | | Where do you usually buy your mobile phone? | | 1.In the village  2.In the town  3.In the county  4.In the city  5.Other____  8.Do not know |  | |
| MP17 | | Does anyone else in your household use a mobile phone? | | 1.Yes  2.No, only I have a mobile phone  3.No, nobody in our household has got a mobile phone  8.Do not know | If MP17=3, go to QQ1 | |
| MP18a | | Regarding the child’s mother:  Does she use a mobile phone? | | 1.Yes  2.No  8.Do not know | If MP18a=2 or 8, go to MP19a | |
| MP18b | | Who owns the mobile phone? | | 1=Herself  2=Father  3=Grandmother 4=Grandfather  5=Other____ | If MP18b=2,3,4,5 go to MP18d1 | |
| MP18c | | Mobile phone number | | _______________________ |  | |
| MP18d1 | | Does she know how to make a phone call with a mobile phone? | | 1.Yes  2.No  8.Do not know |  | |
| MP18d2 | | Does she know how to send a text message? | | 1.Yes  2.No  8.Do not know |  | |
| MP19a | | Regarding the child’s father:  Does he use a mobile phone? | | 1.Yes  2.No  8.Do not know | If MP19a=2 or 8, go to MP20a | |
| MP19b | | Who owns the mobile phone? | | 1=Mother  2=Himself  3=Grandmother 4=Grandfather  5=Other____ | If MP19b=1, 3, 4, or 5 then go to MP19d1 | |
| MP19c | | Mobile phone number | | _______________________ |  | |
| MP19d1 | | Does he know how to make a phone call with a mobile phone? | | 1.Yes  2.No  8.Do not know |  | |
| MP19d2 | | Does he know how to send a text message? | | 1.Yes  2.No  8.Do not know |  | |
| MP20a | | Regarding the grandmother:  Does she use a mobile phone? | | 1.Yes  2.No  8.Do not know | If MP20a=2 or 8, go to question MP21a | |
| MP20b | | Who owns the mobile phone? | | 1=Mother  2=Father  3=Herself  4=Grandfather  5=Other____ | If MP20b=1, 2, 4, or 5 then go to MP20d1 | |
| MP20c | | Mobile phone number | | _______________________ |  | |
| MP20d1 | | Does she know how to make a phone call with a mobile phone? | | 1.Yes  2.No  8.Do not know |  | |
| MP20d2 | | Does she know how to send a text message? | | 1.Yes  2.No  8.Do not know |  | |
| MP21a | | Regarding the grandfather:  Does he use a mobile phone? | | 1.Yes  2.No  8.Do not know | If MP21a=2 or 8, go to MP22a | |
| MP21b | | Who owns the mobile phone? | | 1=Mother  2=Father  3=Grandmother  4=Himself  5=Other____ | If MP21b=1, 2, 3, or 5 go to MP21d1 | |
| MP21c | | Mobile phone number | | _______________________ |  | |
| MP21d1 | | Does he know how to make a phone call with a mobile phone? | | 1.Yes  2.No  8.Do not know |  | |
| MP21d2 | | Does he know how to send a text message? | | 1.Yes  2.No  8.Do not know |  | |
| MP22a | | Are there other adult household members who have interacted frequently with the child? | | 1.Yes  2.No  8.Do not know | If MP22a=2 or 8, go to QQ1 | |
| MP22b | | How does the child refer to him/her? | | ______________________ |  | |
| MP22c | | Does she/he use a mobile phone? | | 1.Yes  2.No  8.Do not know | If MP22c=2 or 8, go to QQ1 | |
| MP22d | | Who owns the mobile phone? | | 1=Mother  2=Father  3=Grandmother 4=Grandfather  5=Him/herself  6=Other____ | If MP22d=1, 2, 3, 4 or 6 go to MP22f1 | |
| MP22e | | Mobile phone number | | _______________________ |  | |
| MP22f1 | | Does she/he know how to make a phone call with a mobile phone? | | 1.Yes  2.No  8.Do not know |  | |
| MP22f2 | | Does she/he know how to send a text message? | | 1.Yes  2.No  8.Do not know |  | |
| **Use of internet (QQ)** | | | | | | |
| QQ1a | | | Do you use your computer in your household to access the Internet? | 1.Yes  2.No  8.Do not know |  | |
| QQ2 | | | Do you use your mobile phone to access the Internet? | 1.Yes  2.No  3.Do not have a mobile phone  8.Do not know |  | |
| **Household (HH)** | | | | | | |
| HH4a | | Is it possible to collect information about the mother? | | | 1.Yes  2.No, remarried  3.No, lost  4.No, passed away |  |
| HH4 | | What is the age of the mother? | | | __________________years  88=Do not know |  |
| HH6 | | What is the mother’s education level? | | | 1.No education  2.Primary school  3.Junior high school  4.Senior high school/technical school  5.Vocational/technical secondary school  6.College  7.University or above  8.Do not know |  |
| HH7 | | How many years of education has the mother had? | | | __________________years |  |
| HH8 | | What is the mother’s occupation? | | | 1.Housework  2.Head of an enterprise, organisation or business unit  3.Technical  4.Receptionist, clerk, secretary  5.Commercial, business and service industry  6.Owner of [individual business](app:lj:%E4%B8%AA%E4%BD%93%E5%B7%A5%E5%95%86%E6%88%B7?ljtype=blng&ljblngcont=0&ljtran=individual%20business)  7.Farmer engaged in non-agricultural industry  8.Industrial workers (factory production worker or transporter, mining, construction) with non-agricultural Hukou  9. Agriculture, forestry, or fishing water conservation  10.Military  11.Other____  88.Do not know |  |
| HH10a | | Is it possible to collect information about the father? | | | 1.Yes  2.No, remarried  3.No, lost  4.No, passed away |  |
| HH10 | | What is the age of the father? | | | __________________years  88=Do not know |  |
| HH12 | | What is the father’s education level? | | | 1.No education  2.Primary school  3.Junior high school  4.Senior high school/technical school  5.Vocational/technical secondary school  6.College  7.University or above  8.Do not know |  |
| HH12a | | How many years of education has the father had? | | | __________________years |  |
| HH13 | | What is the father’s occupation? | | | 1.Housework  2.Head of an enterprise, organisation or business unit  3.Technical  4.Receptionist, clerk, secretary  5.Commercial, business and service industry  6.Owner of [individual business](app:lj:%E4%B8%AA%E4%BD%93%E5%B7%A5%E5%95%86%E6%88%B7?ljtype=blng&ljblngcont=0&ljtran=individual%20business)  7.Farmer engaged in non-agricultural industry  8.Industrial workers (factory production worker or transporter, mining, construction) with non-agricultural Hukou  9. Agriculture, forestry, or fishing water conservation  10.Military  11.Other____  88.Do not know |  |
| HH16a | | What is the age of the primary caregiver? *Only ask if the caregiver is not the mother or the father.* | | | __________________years |  |
| HH16b | | What is the caregiver’s education level? | | | 1.No education  2.Primary school  3.Junior high school  4.Senior high school/technical school  5.Vocational/technical secondary school  6.College  7.University or above  8.Do not know |  |
| HH16c | | How many years’ of education does the caregiver have? | | | __________________years |  |
| HH16d | | What is the caregiver’s occupation? | | | 1.Housework  2.Head of an enterprise, organisation or business unit  3.Technical  4.Receptionist, clerk, secretary  5.Commercial, business and service industry  6.Owner of [individual business](app:lj:%E4%B8%AA%E4%BD%93%E5%B7%A5%E5%95%86%E6%88%B7?ljtype=blng&ljblngcont=0&ljtran=individual%20business)  7.Farmer engaged in non-agricultural industry  8.Industrial workers (factory production worker or transporter, mining, construction) with non-agricultural Hukou  9. Agriculture, forestry, or fishing water conservation  10.Military  11.Other____  88.Do not know |  |
| HH20 | | Family net income in the last year *(after subtracting cost of production)* | | | ________Yuan  888888=Do not know |  |
| HH21 | | Family living expenses in the last year *(living expenses include food, clothing, daily consumable, transport, communication, mortgage/rent, household bills, education, cultural entertainment, hospital bills etc.)* | | | ________Yuan  888888=Do not know |  |

### Survey on demographics and mobile phone use - Chinese version

**家庭编号信息（ID)**

| **ID.1a** | 乡（镇）/街道：___________ | 乡（镇）/街道编号 **ID.1a**□ |
| --- | --- | --- |
| **ID.1b** | 村/居委会：___________ | 村/居委会编号 **ID.1b**□□ |
| **ID.1c** | 儿童编号___________ | 儿童编号 **ID.1c**□□ |
| **ID.2** | 被访者姓名：___________ | **ID.2** |
| **ID.3** | 与儿童的关系： | **ID.3**□ |
|  | 1.母亲 2.父亲 3.（外）祖父母 8．其他：______ |  |
| **ID.4** | 儿童主要养育人是谁？ | **ID.4.1**□ |
|  | 1.母亲 2.父亲 3.（外）祖父母 8．其他：______ |  |
| **ID.5** | 家中6-23月儿童的姓名：_________________ | **ID.5** |
| **ID.6** | 该儿童的性别：1.男 2.女 | **ID.6**□ |
| **ID.7** | 该儿童的出生日期（阳历）：  __ __ __ __年__ __月__ __日 | **ID.7** □□□□ __ _ |
| **ID.8** | 出生体重：__ __g (500g=1斤,50g=1两, *不知道=8888*) | **ID.8**□□□□ |
| **ID.9** | 出生身长：__ __**.**__cm（*不知道=88*） | **ID.9**□□ |
| **ID.10** | 生这个孩子是妈妈第几次怀孕？  __ 次（*如果≥7次，填“7，8.不知道*） | **ID.10**□ |
| **ID.10a** | 妈妈之前生过几个孩子？  __ 次（*如果≥7次，填“7，8.不知道*） | **ID.10a**□ |
| **ID.10b** | 这个孩子是双胞胎或者多胞胎吗？ | **ID.10b**□ |
|  | 1. 否(单胎) 2.双胞胎 3.三胞胎及以上 8.不知道 |  |
| **ID.11** | 母亲生这个孩子的分娩方式？ | **ID.11**□ |
|  | 1.阴道分娩 2.器械助产 3.剖宫产 8.不知道 |  |
| **ID.12** | 这个孩子是怀了多久生的？（例: 38周5天记为38周）  ____周（88.不知道） | **ID.12**□□ |
| **ID.13** | 孩子的户口类型？ 1.城镇户口 2.非城镇户 | **ID.13**□ |
| **ID.14** | 调查员编号___________ | 调查员编号**ID.14**□□ |
| **ID.15** | 调查日期：__ __ __ __年__ __月__ __日 | **ID.15**□□□□5__ __ |

手机使用偏好(MP)

| **MP.1** | 您平时用手机吗？ | | | **MP.1**□ |
| --- | --- | --- | --- | --- |
|  | 1. 用 2. 不用———**转到MP.17** | | |  |
| **MP.2** | 这个手机是什么品牌和型号的？ | | | **MP.2** |
|  | 品牌+型号____________（*8=不知道*） | | |  |
| **MP.3** | 您用的手机是智能手机吗？（展示手机，解释基于安卓、塞班、iOS系统） | | | 1. **MP.3**□ |
|  | 1.是 2.否 8.不知道 | | |  |
| **MP.4** | 当您外出时，您平时用的这个手机经常放在什么地方？ | | | 1. **MP.4**□ |
|  | 1. 自己随身携带 2. 放在家  3. 其他人带着手机 4. 其他___________ | | |  |
| **MP.5** | 您主要是用手机打电话还是发短信？ | | | **MP.5**□ |
|  | 1.电话 2. 短信 3. 差不多 4.其他_________ | | |  |
| **MP.5a** | 您平常习惯使用手机上QQ还是发短信？ | | | **MP.5a**□ |
|  | 1.QQ 2.短信 3.差不多 4.都不会用 5.其他_________ 8.不知道 | | |  |
| **MP.6** | 您平均每周拨出电话的数量？______ (88.不知道 99.不会打电话) | | | **MP.6**□□ |
| **MP.7** | 您平均每周接到电话的数量？______ (88.不知道 99.不会接电话) | | | **MP.7**□□ |
| **MP.8** | 您平均每周发出短信的数量？______ (88.不知道 99.不会发短信) | | | **MP.8**□□ |
| **MP.9** | 您平均每周收到短信的数量？______ (88.不知道 99.不会收短信) | | | **MP.9**□□ |
| **MP.10** | 您用的这部手机平均每个月的话费是多少钱？ _________元（888.不知道） | | | **MP.10**□□□ |
| **MP.11** | 您过去一年更换过几次电话号码？ | | | **MP.11**□ |
|  | 1. 没有换过 2. 换过1次 3. 换过2次 4.换过3次  5. 换过4次及以上 8. 不记得 | | |  |
| **MP.12** | 现在手机是否能正常使用？（指手机的性能,包括因欠费导致的功能不正常） | | | **MP.12**□ |
|  | 1.能打电话和发短信 2.仅能打电话不能发短信 3.仅能发短信不能打电话 4.不能打电话也不能发短信 5.其他状况_________ | | |  |
| **MP.13** | 您家里面有人有下列人员的的电话号码吗？（包括座机和手机） | | |  |
|  | 1.县级医院或以上  （包括县级医院，县妇幼保健院，私立医院） | | 1．是 2.否 8.不知道 | **MP.13.a**□□ |
|  | 2.乡镇卫生院 | | 1．是 2.否 8.不知道 | **MP.13.b**□□ |
|  | 3.村卫生室 | | 1．是 2.否 8.不知道 | **MP.13.c**□□ |
| **MP.14** | 在过去的3个月中，您有几次通过手机得到关于您自己或孩子健康方面的相关信息？（短信和电话都算） | | | **MP.14**□ |
|  | 1.从来没有过 2.一次— **转到MP.16** 3. 两次——两**转到MP.16**  4. 三次—— **转到MP.16** 5.大于三次——大**转到MP.16** | | |  |
| **MP.15** | 你是否愿意通过手机获得健康信息？ | | | **MP.15**□ |
|  | 1.是 2.否 3.其他 | | |  |
| **MP.16** | 您一般在哪里购买手机？ | | | **MP.16**□ |
|  | 1.村附近 2.镇上 3.县城里 4.市区 5.其他_______ 6.不知道 | | |  |
| **MP.17** | 您家里的其他人平时用手机吗？ | | | **MP.17**□ |
|  | 1. 用 2.家里只有我用手机  3 家里所有人都不用手机—— **转到QQ.1**  8.不知道其他人的状况 | | |  |
| **MP.18.a** | **母亲**有用手机的习惯吗？ | | | **MP.18.a**□ |
|  | 1．有 2.没有——没**转到MP.19.a** 8.不知道—— **转到MP.19a** | | |  |
| **MP.18.b** | 母亲使用的是谁的手机？ | | | **MP.18.b**□ |
|  | 1.母亲 2.父亲— **转到MP.18.d1** 3. 奶奶（姥姥）— **转到MP.18.d1**  4.爷爷（姥爷）—**转到MP.18.d1** 5.其他— **转到MP.18.d1** | | |  |
| **MP.18.c** | 母亲手机的电话号码 (88. 不知道 ) | | | **MP.18.c**□□□□□□□□□□□ |
|  | 母亲能够用手机完成 | | |  |
| **MP.18.d1** | 打电话 | 1．能 2.不能 8.不知道 | | **MP.18.d1**□ |
| **MP.18.d2** | 发短信 | 1．能 2.不能 8.不知道 | | **MP.18.d2**□ |
| **MP.19.a** | **父亲**有用手机的习惯吗？ | | | **MP.19.a**□ |
|  | 1．有 2.没有——**转到MP.20.a** 8.不知道——**转到MP.20.a** | | |  |
| **MP.19.b** | 父亲使用的是谁的手机？ | | | **MP.19.b**□ |
|  | 1.母亲—.**转到MP.19.d** 2.父亲  3. 奶奶（姥姥）—**转到MP.19.d1** 4.爷爷（姥爷）—**转到MP.19.d**1  5.其他—**转到MP.19.d1** | | |  |
| **MP.19.c** | 父亲手机的电话号码 (88. 不知道 ) | | | **MP.19.c**□□□□□□□□□□□ |
|  | 父亲能够用手机完成 | | |  |
| **MP.19.d1** | 打电话 | 1．能 2.不能 8.不知道 | | **MP.19.d1**□ |
| **MP.19.d2** | 发短信 | 1．能 2.不能 8.不知道 | | **MP.19.d2**□ |
| **MP.20.a** | **奶奶/姥姥**有用手机的习惯吗？ | | | **MP.20.a**□ |
|  | 1．有 2.没有——没**转到MP.21.a** 8.不知道——**转到MP.21.a** | | |  |
| **MP.20.b** | 奶奶/姥姥使用的是谁的手机？ | | | **MP.20.b**□ |
|  | 1. 母亲—.**转到MP.20.d1** 2.父亲— **转到MP.20.d1** 3.奶奶（姥姥）  4.爷爷（姥爷）—**转到MP.20.d1**  5.其他—**转到MP.20.d1** | | |  |
| **MP.20.c** | 奶奶/姥姥所使用手机的电话号码 （88.不知道） | | | **MP.20.c**□□□□□□□□□□□ |
|  | 奶奶/姥姥能够用手机完成 | | |  |
| **MP.20.d1** | 打电话 | 1．能 2.不能 8.不知道 | | **MP.20.d1**□ |
| **MP.20.d2** | 发短信 | 1．能 2.不能 8.不知道 | | **MP.20.d2**□ |
| **MP.21.a** | **爷爷/姥爷**有用手机的习惯吗？ | | | **MP.21.a**□ |
|  | 1．有 2.没有——**转到MP.22.a** 8.不知道——**转到MP.22.a** | | |  |
| **MP.21.b** | 爷爷/姥爷使用的是谁的手机？ | | | **MP.21.b**□ |
|  | 1.母亲—**转到MP.21.d1** 2.父亲— **转到MP.21.d1**  3.奶奶（姥姥）—.**转到MP.21.d1**） 4.爷爷（姥爷）5.其他—.**转到MP.21.d1** | | |  |
| **MP.21.c** | 爷爷/姥爷所使用手机的电话号码 （88. 不知道） | | | **MP.21.c**□□□□□□□□□□□ |
|  | 爷爷/姥爷能够用手机完成 | | |  |
| **MP.21.d1** | 打电话 | 1．能 2.不能 8.不知道 | | **MP.21.d1**□ |
| **MP.21.d2** | 发短信 | 1．能 2.不能 8.不知道 | | **MP.21.d2**□ |
| **MP.22.a** | **家中还有其他大人**和孩子长期在一起吗？ | | | **MP.22.a**□ |
|  | 1.有 2.没有——没**转到QQ.1** 8.不知道——**转到QQ.1** | | |  |
| **MP.22.b** | 孩子对他/她的称呼是？ ________ 88.不知道 | | | **MP.22.b** |
| **MP.22.c** | 他/她有用手机的习惯吗？ | | | **MP.22.c**□ |
|  | 1．有 2.没有——没**转到QQ.1** 8.不知道——8**转到QQ.1** | | |  |
| **MP.22.d** | 他/她使用的是谁的手机？ | | | **MP.22.d**□ |
|  | 1.母亲—**转到MP.22.f1** 2.父亲— **转到MP.22.f1**  3.奶奶（姥姥）—**转到MP.22.f1** 4.爷爷（姥爷）—姥**转到MP.22.f1**  5.自己 6.其他—.**转到MP.22.f1** | | |  |
| **MP.22.e** | 他/她所使用手机的电话号码 （88. 不知道） | | | **MP.22.e**□□□□□□□□□□□ |
|  | 他/她能够用手机完成 | | |  |
| **MP.22.f1** | 打电话 | 1．能 2.不能 8.不知道 | | **MP.22.f1**□ |
| **MP.22.f2** | 发短信 | 1．能 2.不能 8.不知道 | | **MP.22.f2**□ |
| **QQ使用偏好(QQ)** | | | | |
| **QQ.1a** | 你用家里电脑上网吗？ | | | **QQ.1a**□ |
|  | 1.用  2.不用  8.不知道 | | |  |
| **QQ.2** | 你用手机上网吗？ | | | **QQ.2**□ |
|  | 1.用  2.不用  3.没有手机  8.不知道 | | |  |

**家庭基本信息（HH)**

| **HH.4a** | 是否可以记录母亲信息 | | | **HH.4a**□ |
| --- | --- | --- | --- | --- |
|  | 1.可以 2.再婚***—— 转到HH.10a***  3.下落不明***——不转到HH.10a*** 4.去世***——.转到HH.10 a*** | | |  |
| **HH.4** | 母亲的年龄：__ __周岁 （88-不知道） | | | **HH.4a**□□ |
| **HH.6** | 母亲的文化程度 | | | **HH.6**□ |
|  | 1.没上过学***——没转到HH.8***  2.小学  3.初中  4.高中/技校 | | 5. 中专/中技  6. 大专  7. 大学以上  8. 不清楚 |  |
| **HH.7** | 母亲上了多少年学？ | | | **HH.7**□□ |
|  | __ __ 年 （0.没有上过学，88. 不知道） | | |  |
| **HH.8** | 母亲的工作 | | | **HH.8**□ |
|  | 1.家务  2.机关、企事业单位负责人  3.专业技术人员  4.一般办事人员  5.商业服务业人员  6.个体工商户 | | 7.从事非农劳动的农民  8.非农业户口的产业工人  9. 农林牧渔水利业生产人员  10.军人  11.其他____________________  88.不知道 |  |
| **HH.10a** | 是否可以记录父亲信息 | | | **HH.10a**□ |
|  | 1.可以 2.再婚***—— 转到HH.15***  3.下落不明***——不转到HH.15*** 4.去世***——.转到HH.15*** | | |  |
| **HH.10** | 父亲年龄 __ __周岁（88-不知道） | | | **HH.10**□□ |
| **HH.12** | 父亲的文化程度 | | | **HH.12**□ |
|  | 1.没上过学***——没转到HH.13***  2.小学  3.初中  4.高中/技校 | | 5. 中专/中技  6. 大专  7. 大学以上  8. 不清楚 |  |
| **HH.12a** | 父亲上了多少年学？ | | | **HH.12a**□□ |
|  | __ __ 年 （0.没有上过学，88. 不知道） | | |  |
| **HH.13** | 父亲的工作 | | | **HH.13**□ |
|  | 1.家务  2.机关、企事业单位负责人  3.专业技术人员  4.一般办事人员  5.商业服务业人员  6.个体工商户 | 7.从事非农劳动的农民  8.非农业户口的产业工人  9. 农林牧渔水利业生产人员  10.军人  11.其他____________________  88.不知道 | |  |
| **HH.16a** | 父母以外的主要养育人年龄？ __ __ 周岁 | | | **HH.16a**□□ |
| **HH.16b** | 父母以外的主要养育人的文化程度 | | | **HH.16b**□ |
|  | 1.没上过学***——没转到HH.16d***  2.小学  3.初中  4.高中/技校 | 5. 中专/中技  6. 大专  7. 大学以上  8. 不知道 | |  |
| **HH.16c** | 父母以外的主要养育人上了多少年学？ | | | **HH.16c**□□ |
|  | __ __ 年 （0.没有上过学，88. 不知道） | | |  |
| **HH.16d** | 父母以外的主要养育人的职业 | | | **HH.16d**□ |
|  | 1.家务  2.机关、企事业单位负责人  3.专业技术人员  4.一般办事人员  5.商业服务业人员  6.个体工商户 | 7.从事非农劳动的农民  8.非农业户口的产业工人  9. 农林牧渔水利业生产人员  10.军人  11.其他____________________  88.不知道 | |  |
| **HH.20** | 您的家庭去年纯收入为（元）__ __ __ __ __ __（*家庭去年扣去生产成本后各种收入的总和*）不知道填”888888” | | | **HH.20**□□□□□□ |
| **HH.21** | 您的家庭去年生活消费性支出为__ __ __ __ __ __（元）  不知道填”888888”  （生活性消费支出包括食品、衣着、日用品、交通、通讯、住房、水电及燃料、教育、文化娱乐、医疗等方面的支出） | | | **HH.21**□□□□□□ |

###

### Topic guides for semi-structured interviews with caregivers

***Topic guide 1 (first round of interviews)***

| **Research questions**   1. How do people use a mobile phone? 2. What is people’s experience with seeking information for their child’s health? |
| --- |

***GENERAL SECTION.***

***To start off, I would like to find out a little about you and your family:***

1. What do you usually do during the day?
2. Can you please describe your family members and their relationship to you?
3. Who will take care of the child when he or she is ill?

***SECTION 1.***

***We will continue to talk about your mobile phone use:***

1. Can you show me your mobile phone? *(if participant cannot show the mobile phone ask what sort of mobile phone it is)* Do you use any other mobile phones?
2. Where do you keep your mobile phone?
3. Why do you use a mobile phone?
4. Which functions of the mobile phone do you use?
5. Which do you prefer: sending text messages or making phone calls? Why?
6. Sometimes people have problems with using their mobile phone. For example a phone stops working, someone deletes a text message accidently by pressing the wrong button, or someone realizes that there is not enough credit on the phone. Have you ever seen something like this happening?
7. How much money do you spend on using your mobile phone?
8. How long have you used a mobile phone?
9. How often do you change your mobile phone number? Why?
10. Did you purchase this mobile phone? If not, who purchased the mobile phone? On what occasion did he or she give you the mobile phone?
11. Can you tell me about other people in your household using a mobile phone?

***Before I ask you more questions, I just want to briefly sum what you told me about using your mobile phone. You said………………, is that right?***

***SECTION 2.***

***I would now like to ask you a bit about seeking information or advice when your child has a health problem:***

1. Who do you first ask for help when your child has a health problem?
2. Have you sought any information about your child’s health from a doctor working in a village clinic or township hospital in the past year? Please tell me about the most recent time you sought information about your child’s health.
3. Have you contacted a doctor working in a village clinic or township hospital by a mobile phone in the past year? Please tell me about your most recent contact with a health worker via your mobile phone.
4. Do you use health-related applications on your mobile phone? If so, please tell me more about this.
5. Would you be interested in participating in follow-up research? *(if so, please record contact details carefully)*

***Before we end this interview, I just want to briefly sum what you told me about seeking health information. You said………………, is that right?***

***Thank you very much for taking part (stress confidentiality and anonymity again).Topic guide 2 (second round of interviews)***

| **Research questions**   1. Which factors influence whether people respond to text messages? 2. What is people’s experience with seeking information for their child’s health via their mobile phone? |
| --- |

***SECTION 1.***

***We will talk about your mobile phone use:***

1. When did you use a mobile phone for the first time?
2. Why do you use a mobile phone?
3. Which functions of the mobile phone do you use?
4. Which function do you like best? Why?
5. Now I would like to talk more about mobile phone text messaging. How long does it normally take for you to notice receiving a text message?
6. When you notice you have received a text message, when will you respond to it?
7. What kind of messages do you respond to? What kind of messages do you ignore? Why?
8. Have you ever received information or questions via mobile phone text messages? If so, please tell me more about this.

***Before I ask you more questions, I just want to briefly sum what you told me about using your mobile phone and text messaging. You said………………, is that right?***

***SECTION 2.***

***I would now like to ask you a bit about seeking information or advice when your child has a health problem:***

1. Who do you first ask for help when your child has a health problem?
2. Have you sought any information about your child’s health from a doctor working in a village clinic or township hospital in the past year? Please tell me about the most recent time you sought information about your child’s health.
3. Have you contacted a doctor working in a village clinic or township hospital by a mobile phone in the past year? Please tell me about your most recent contact with a health worker via your mobile phone.
4. Do you use health-related applications on your mobile phone? If so, please tell me more about this.
5. Have you sought any health information on the internet? If so, please tell me more about this.
6. Would you be interested in participating in follow-up research? *(if so, please record contact details carefully)*

***Before we end this interview, I just want to briefly sum what you told me about seeking health information. You said………………, is that right?***

***Thank you very much for taking part (stress confidentiality and anonymity again).***
