# Supplementary material for: Prevalence of Mobile Phones and Factors Influencing Usage by Caregivers of Young Children in Daily Life and for Health Care in Rural China: A Mixed Methods Study
Source: PLoS One. 2015 Mar 19;10(3):e0116216. doi: 10.1371/journal.pone.0116216 (PMC4366174; doi:10.1371/journal.pone.0116216)
Supplement: S1 Table — (DOCX) [file pone.0116216.s002.docx]

**Table S1**

**Survey participants’ and household members' mobile phone usage (N=1854)**

|  | **Mothers** | |  | **Fathers** | |  | **Grand**  **mothers** | |  | **Grandfathers** | |  | **Other caregivers** | |
| --- | --- | --- | --- | --- | --- | --- | --- | --- | --- | --- | --- | --- | --- | --- |
|  | **n (%)** | **N^a^** |  | **n (%)** | **N^b^** |  | **n (%)** | **N^b^** |  | **n (%)** | **N^†^** |  | **n (%)** | **N** |
|  |  |  |  |  |  |  |  |  |  |  |  |  |  |  |
| **Using mobile phone** |  |  |  |  |  |  |  |  |  |  |  |  |  |  |
| Yes | 1666 (89.9) | 1853 |  | 1758 (94.9) | 1853 |  | 743 (40.1) | 1853 |  | 876 (47.3) | 1852 |  | 123 (41.6) | 296 |
| No | 178  (9.6) |  |  | 79 (4.3) |  |  | 1082 (58.4) |  |  | 949 (51.2) |  |  | 163 (55.1) |  |
| Do not know | 9 (0.5) |  |  | 15 (0.8) |  |  | 27 (1.5) |  |  | 27 (1.5) |  |  | 10 (3.3) |  |
| **Owners of mobile phone** |  |  |  |  |  |  |  |  |  |  |  |  |  |  |
| Mothers | 1653 (99.2) | 1666 |  | 25 (1.4) | 1758 |  | 7 (1.0) | 743 |  | 1  (0.1) | 876 |  | 1 (0.8) | 123 |
| Fathers | 11 (0.7) |  |  | 1731 (98.5) |  |  | 10 (1.3) |  |  | 13 (1.5) |  |  | 1 (0.8) |  |
| Grand  mothers | 0 (0.0) |  |  | 0 (0.0) |  |  | 712 (95.8) |  |  | 12 (1.4) |  |  | 1 (0.8) |  |
| Grand  fathers | 0 (0.0) |  |  | 0 (0.0) |  |  | 13 (1.7) |  |  | 850 (97.0) |  |  | 6 (4.9) |  |
| Other caregivers | 2 (0.1) |  |  | 2 (0.1) |  |  | 1 (0.2) |  |  | 0  (0.0) |  |  | 113 (91.9) |  |
| Other person | 0 (0.0) |  |  | 0 (0.0) |  |  | 0 (0.0) |  |  | 0  (0.0) |  |  | 1 (0.8) |  |
| **Able to make phone call** |  |  |  |  |  |  |  |  |  |  |  |  |  |  |
| Yes | 1645  (98.8) | 1666 |  | 1734 (98.6) | 1758 |  | 718 (96.6) | 743 |  | 857 (97.8) | 876 |  | 121 (98.4) | 123 |
| No | 18 (1.1) |  |  | 22 (1.3) |  |  | 23 (3.1) |  |  | 14 (1.6) |  |  | 2  (1.6) |  |
| Do not know | 2 (0.1) |  |  | 2 (0.1) |  |  | 2 (0.3) |  |  | 5  (0.6) |  |  |  |  |
| **Able to send text message** |  |  |  |  |  |  |  |  |  |  |  |  |  |  |
| Yes | 1505 (90.4) | 1666 |  | 1624 (92.4) | 1758 |  | 171 (23.0) | 743 |  | 264 (30.1) | 876 |  | 96 (78.0) | 123 |
| No | 157 (9.4) |  |  | 130 (7.4) |  |  | 568 (76.4) |  |  | 601 (68.6) |  |  | 27 (22.0) |  |
| Do not know | 3 (0.2) |  |  | 4 (0.2) |  |  | 4 (0.6) |  |  | 11 (1.3) |  |  | 0  (0.00) |  |

^a^One mother discontinued the interview and did not answer this question.

^b^Two mothers discontinued the interview and did not answer these questions.
