# Supplementary material for: Prevalence of Mobile Phones and Factors Influencing Usage by Caregivers of Young Children in Daily Life and for Health Care in Rural China: A Mixed Methods Study
Source: PLoS One. 2015 Mar 19;10(3):e0116216. doi: 10.1371/journal.pone.0116216 (PMC4366174; doi:10.1371/journal.pone.0116216)
Supplement: S1 Text — (DOCX) [file pone.0116216.s003.docx]

**Text S1**

### Theme 2: Usage of mobile phone functions

#### Problems whilst using mobile phones

The following problems that occurred whilst using mobile phones are described: (i) non-functioning mobile phone; (ii) phone running out of credit; (iii) empty battery; (iv) lost mobile phone; and (v) radiation.

##### Not functioning mobile phone

Sometimes mobile phones did not function because of accidents such as dropping the mobile phone in water (I5, 8). In other cases, mobile phones did not work well without a clearly identifiable reason (I4, 6, 7, 9)*.* It was thought that mobile network problems caused the mobile phone to dysfunction, but it was not always known for sure whether this was a mobile phone or network problem (I9). When possible, caregivers solved mobile phone problems themselves (I4). When caregivers could not solve problems themselves, they would take their mobile phone to a repair shop or buy a new mobile phone (I4, 8).

##### Running out of credit

It occasionally happened that there was no mobile phone credit left on the mobile phone. Depending mobile telecom operator policies and user age and credit, some could still use the mobile phone when there was minus ¥2 (about £0.20) or more credit in debt. Still, caregivers had to be careful with recharging credit before their mobile phone credit ran out, because otherwise they were unable to use their mobile phone. When having a family, it was important to stay in touch and arrangements were sometimes made to recharge credit for the whole family (I2). Text message reminders were received when mobile phone credit was low (I2, 3, 7, 9). Credit could be bought in the mobile telecom operator service centre (I1, 2), but being able to buy credit online was found convenient (I1, 9).

##### Battery runs out

Sometimes the mobile phone ran out of battery (I1, 3), but usually mobile phones were charged before this occurred (later described as *Mobile phone switched* *on* in Supporting Information 4).

##### Losing mobile phone

Sometimes a mobile phone was lost and then a new mobile phone was bought (I4, I9). In these cases, money had to be paid if someone wanted to keep the original mobile phone number, which was infrequently done. Therefore, it was more common to change the mobile phone number when a mobile phone was lost (I4).

##### Radiation from mobile phone

Radiation from mobile phones, computers and televisions was perceived as harmful and spontaneously mentioned by a number of caregivers (I1, 2, 7, 8, 12, 13). This information was obtained from relatives, friends, books, newspapers, magazines and television (I1, 7, 8, 12, 13). Radiation was thought to be particularly harmful to infants, young children and pregnant women (I1, 2, 12). An observed effect of radiation was that a child got fever when using the computer or when playing with the mobile phone (I13). The harmful effects of radiation were not always very clear to caregivers, but it was thought that exposure to radiation could lead to malformation during child development (I1). Despite the perceived harmful effects of mobile phone radiation, still mobile phones were used. The radiation was not so bad, because preventive measures could be taken that limited the harmful effects of radiation (I1).

*Mother: “I just feel that I can live with the radiation, because the radiation is something that you can try to keep away. Yes, just that, when you are careful the radiation will not do big harm to the body. You can just be careful to avoid those occasions, using a mobile phone when the radiation is strong. It's OK if you are careful, the radiation, actually, is not a big harm...though I pay attention to radiation, but I don't think it's a big problem”. “*就是感觉……就是这个辐射就是还可以接受的的情况，因为这个辐射毕竟…就是说，他就是你可以避免或者就是可以减…减少对，对，就是那个，平时注意的话，他这个辐射对身体，比如说，没有太大危害，就是说，你平时注意不要…就是说这种情况下，容易辐射比较高的情况下使用，就是，都可以。所以说你平时注意就行了，他这个辐射其实没有太…虽然说就是重视辐射，但是没有把它想象的太严重*”*。 *(I1)*

When having a child, it was more important to pay attention to radiation and the mobile phone was often kept away from the child (I13). Other preventive measures included not using the mobile phone when being pregnant (I2, 12) and reducing the duration of mobile phone use (I1). The perceived effects of mobile phone radiation played a role in the place where the mobile phone was kept. Sometimes the mobile phone was not kept in the pocket (I12). Also the mobile phone was kept in places further away from people, such as near the window (I7) or on the table during the night (I1, 8). For reducing radiation from computers, a cactus was placed next to the computer which was though to weaken the radiation by absorbing it (I1).
