# Supplementary material for: Prevalence of Mobile Phones and Factors Influencing Usage by Caregivers of Young Children in Daily Life and for Health Care in Rural China: A Mixed Methods Study
Source: PLoS One. 2015 Mar 19;10(3):e0116216. doi: 10.1371/journal.pone.0116216 (PMC4366174; doi:10.1371/journal.pone.0116216)
Supplement: S2 Text — (DOCX) [file pone.0116216.s004.docx]

**Text S2**

### Theme 2: Usage of mobile phone functions

#### Comparison between phone calls and sending text messages

The following factors related to preferences are described: (i) immediacy; (ii) clarity; (iii) ease of use; and (iv) costs.

##### Immediacy

Making a phone call as was quicker, because communication via text messaging took a longer time (I2, 3, 5, 15). When the mobile network did not function well, the text message could be delayed and waiting for a text message reply was found to take a long time (I1).

*Mother: “Sometimes, it is clearer to talk; text messages… sometimes text message is not as quick as making phone calls. I mean…I mean, for communication, text message is a two-way process (laughing with sounds), like sending messages repeatedly, and wait… wait for the other’s response. Now if you need… you can make a phone call to say it. Right?”* “有的时候话说的明白，短信…而且有时候发短信不如打电话及时，就是…就是沟通是吧，短信还有一个来回，（笑）反复发送啊，等…等对方回信息.现在有什么事儿打电话就说了是吧*”*。*(I1)*

Phone calls were found to be more direct (I2, 3). Therefore, in urgent situations calling was preferred over sending a text message (I12). However, when being busy with work, it was better to receive a text message that could be read later. A text message was also sent when someone could not be reached via a phone call (I9).

##### Clarity

Communicating by making phone calls was found to be clearer than sending text messages (I1, 3, 6-8, 10, 15). Text messages only had a limited space and thus it was more difficult to make matters be clearly understood (I6, 14, 15). Conversely, text messaging was preferred when it was found difficult to express something in a phone calls and clearer to write (I9).

##### Ease of use

It was found too much effort to type a text message (I1, 6, 10, 15). Writing a text message took a long time. Caregivers did not have the patience to send text messages or the child asked for caregivers’ attention whilst writing a text message (I3, 10).

Even when text messaging was preferred, still it was found uneasy to write text messages. This was caused by the Chinese input methods (Pinyin), which were perceived to be more difficult to use compared to input methods based on the alphabet (I9).

*Mother: “Because it is not like the….because my mobile phone does not have the input method for directly tracing the characters (does not have mobile phone with touch screen). I think it is faster to write/trace them. Input is only via pinyin typing… Pinyin… It is not the same as there are 26 symbols for 26 letters. There are many letters on one button, it is too slow to type”. “因为她不是像那个，因为我这手机没有手写功能嘛，写的时候可能比较快一些，就是得拼写嘛，拼写，也不是26个字母都有26个字符表示，它一个键上有好几个字母得找那个太慢了”。(I9)*

##### Costs

Costs were a noticeable consideration. Calling could save money compared to sending text messages (I8, 13). A short phone call was preferred when contacting someone local, because this was cheaper than sending multiple text messages. Local calls were cheaper than long-distance calls (I13, 15). However, when the person who received the call was in a different region, a text message was sent to save money (I15).

*Father: “I’m not used to it and I think it is too much effort (text messaging). Unless I am far away from home, when I need to make a long distance call, I will send text messages”. “就是不习惯回短信。嫌回个麻烦，除非出远门了在外边，打电话打长途了干嘛了，回个短信”。(I15)*
