# Supplementary material for: Prevalence of Mobile Phones and Factors Influencing Usage by Caregivers of Young Children in Daily Life and for Health Care in Rural China: A Mixed Methods Study
Source: PLoS One. 2015 Mar 19;10(3):e0116216. doi: 10.1371/journal.pone.0116216 (PMC4366174; doi:10.1371/journal.pone.0116216)
Supplement: S3 Text — (DOCX) [file pone.0116216.s005.docx]

**Text S3**

### Theme 3: Replying to text messages

#### Checking mobile phones

The following factors related to checking the mobile phone are described: (i) when the user is free; (ii) where the mobile phone is placed; (iii) mobile phone switched on; and (iv) mobile phone audio volume.

##### When the user is free

Checking the mobile phone depended on the available time; usually the mobile phone was checked during free time and when it was not busy (I4, 10-12). Free time was when the child was asleep in the afternoon or evening. When taking care of the child, the mobile phone was often not checked (I10).

##### Where the mobile phone is placed

Table 6 shows that almost all surveyed fathers carried their mobile phone with them when leaving the house (40; 97.6%), but only about two third of mothers (975; 68.0%) and grandparents (96; 67.6%) carried their mobile phones outside the home.

The place where the mobile was kept varied for semi-structured interview participants. The mobile phone was often brought when going out, if it was not forgotten. When being home, the mobile phone was often kept at a particular place in the house (I1-3, 5, 6, 8, 10, 11-13, 15).

*Mother： “Eh…I put it on the table at home. I keep the mobile phone in my pocket when I go out”. “恩…… 在家的时候放桌子上，在外边一般是放在那口袋吧”。(I8)*

Sometimes it was forgotten to take the mobile phone out of the pocket at home (I9) or the mobile phone was purposely carried by the caregiver (I4, 14). Where caregivers shared a mobile phone, the person going out was given the phone (I2).

##### Mobile phone switched on

Usually the mobile phone was kept switched on during both day and night (I1-3). Work sometimes required that a caregiver had to be contactable at any time. Even when there was no work requirement to keep the mobile phone switched on, it was left on in case of emergencies or when family members called. Only when the mobile phone was out of battery was it turned off (I1).

##### Mobile phone audio volume

Usually the sound of the mobile phone was on and an incoming phone call or text message could be heard (I2-8, 10, 11). The sound was even kept on during the night for when something happened (I3). However, the sound was not always very clear when being further away from the mobile phone (I6) and less attention was paid to the mobile phone when taking care of the child (I10).

*Mother: “I can hear it…sometimes when my child needs me or my child is naughty, I do not care about that, I will take care of my child first (laughing with sounds) ”. “听见…有时候孩子正需要人咧，正淘呢，就不管那个了，就是以孩子为主先（妈妈笑了）” (I10)*
